# Multireference diffusion Monte Carlo reaches 2D materials

**DOI:** 10.1038/s41598-025-17564-3

**Published:** 2025-09-26

**Authors:** Nicole Spanedda, Anouar Benali, Fernando A. Reboredo, Jaron T. Krogel

**Affiliations:** 1 Materials Science and Technology Division, Oak Ridge National Laboratory, Oak Ridge, USA; 2 Computational Science Division, Argonne National Laboratory, Lemont, USA

**Keywords:** Quantum physics, Condensed-matter physics, Electronic properties and materials

## Abstract

Quantum confinement in 2D materials strongly enhances electronic correlation effects. Therefore, predicting the properties of these unique materials, with both a high level of accuracy and computational efficiency, without relying on adjustable parameters or functionals, remains an outstanding theoretical challenge. The majority of theoretical studies are based on the approximations of density functional theory (DFT). The reliability of DFT predictions are heavily dependent on the choice of an approximated exchange-correlation functional. Here, we estimate the magnitude of impact of correlation on the total energy for the quintessential 2D material, graphene, by performing and comparing state-of-the-art selected CI and quantum Monte Carlo extrapolated calculations for a single unit cell at the $\Gamma$ point. We demonstrate that Self-Healing Diffusion Monte Carlo (SHDMC) obtains a very compact, but high-quality wavefunction for this system that lacks the strong basis set dependence displayed by state of the art quantum chemistry methods. The SHDMC wavefunction is of higher quality compared to that obtained from sCI, in the same orbital basis, while being $\sim$ 1000 times smaller in terms of determinant count compared to sCI. We also demonstrate that extrapolating SHDMC results to the infinite determinant limit compares extremely well with complete basis set extrapolated sCI. Our work paves the way for future validation of SHDMC applied to challenging 2D materials.

## Introduction

The understanding and design of novel 2D materials requires the inclusion of correlated many-body effects within accurate theoretical and computational methodologies in order to predict and assist in the interpretation of novel experiments. This goal requires overcoming the limitations of density functional theory (DFT)^1,2^, which is one of the most commonly used approaches for computationally predicting the properties of materials, including 2D materials. Although vdW DFT functionals have improved the prediction of the properties of 2D layered materials, which display van der Waals bonding, it has been demonstrated that results obtained can display high sensitivity to the choice of the vdW functional^3–5^.

Correlated many-body effects could be accounted for with quantum Chemistry approaches. A variety of coupled-cluster based schemes have been applied to predict the properties of real materials, including 2D materials, with high levels of accuracy but display a very steep scaling of computational cost with respect to system size^6,7^.

Essential information about materials including their stability can be obtained through ground state properties, such as the total ground state energy. It has been demonstrated that Diffusion Monte Carlo (DMC) is an accurate and reliable option for calculating ground state properties of 2D materials^8–10^. DMC is stochastic projector based method that allows the calculation of ground state properties of a system of interest by sampling its ground state wave function.

An unrestricted DMC approach converges to a ground state wavefunction that is bosonic, which is problematic when attempting to predict the properties of fermionic systems^11^. This problem is well documented and it is a manifestation of the fermion sign problem in DMC. The most widely used approach for effectively handling the fermion sign problem in Quantum Monte Carlo (QMC) methods is the fixed-node approximation^12^. Typically, the DMC algorithm for fermions utilizes importance sampling, where the importance distribution is the probability density of a chosen fermionic trial wavefunction^13^. The fixed-node approximation works by enforcing that the ground state wavefunction has the same nodes as the chosen trial wavefunction^14,15^. As a consequence, fixed-node DMC (FN-DMC) finds the lowest energy wavefunction that has the same nodal surface as the given trial wavefunction. Fixed-node DMC is capable of finding the ground state of a given system, but only if the nodal surface of the chosen trial wavefunction exactly matches the nodal surface of the ground state wavefunction. Otherwise DMC has a positive bias in the total energy, which is denoted as the nodal error. As a result, the choice of the trial wavefunction is imperative for predictions of the ground state energetics of materials, with an accuracy comparable to that of quantum chemistry methods. One of the advantages that FN-DMC displays over quantum chemistry methods is that it displays significantly less dependence on which orbital basis set is used^16,17^.

A common choice for the trial wavefunction in solid-state systems is to use a single determinant Slater-Jastrow trial wavefunction with orbitals obtained from either DFT or Hartree-Fock^18^. It has been demonstrated that FN-DMC calculations using DFT based trial wavefunctions can improve the accuracy of predicted properties of materials compared with DFT alone. For example, it has been found that FN-DMC can more accurately predict a variety of properties, including but not limited to, cohesive energies, phase stability, vacancy formation energies, lattice constants, and band gaps of correlated materials compared to DFT^19–24^. It has also been demonstrated that FN-DMC can more accurately predict the ground-state energies and temperature-dependent Helmholtz free energies of both the perovskite and post-perovskite phases of $\textrm{MgSi}\textrm{O}_{3}$ compared to DFT^25^. Though generally small, it has been shown that the single-determinant fixed-node error can significantly impact the results obtained for a variety of many-electron systems^26–29^.

Although single-determinant trial wavefunctions often perform reasonably well for some systems, it has been demonstrated that alternative choices, such as multideterminant trials wavefunctions, can provide more accurate results. For example, it has been demonstrated that using trial wavefunctions from selected configuration interaction (sCI) methods can enhance the accuracy of the results obtained with FN-DMC for molecules, clusters, and solids^30–33^. However, sCI typically generates trial wavefunctions containing a vast number of terms which rapidly becomes impractical when investigating materials that have a large number of electrons.

Within the DMC approach, a projected gradient descent has been used to iteratively move the nodes in a direction that was argued to lower the DMC energy by systematically updating the parameters of the anti-symmetric part of the wavefunction^34^.

A promising approach that is capable of directly optimizing the nodes of the wavefunction within the DMC regime is Self-Healing Diffusion Monte Carlo (SHDMC)^35–37^. SHDMC is an iterative procedure that gradually moves the nodes of an input trial wave function towards the nodes of the ground-state wavefunction. An advantage of SHDMC is that the results obtained are independent of the choice of the initial trial wavefunction^35,36^. The utility of SHDMC has previously been demonstrated for investigating atoms (oxygen), diatomic molecules ($\mathrm {N_{2}}$), and clusters ($\mathrm {C_{20}^{+2}}$)^36^.

In this work, we demonstrate that SHDMC is capable of estimating the DMC fixed-node error in graphene (unit cell at the $\Gamma$ point), as an example 2D material. We demonstrate that SHDMC can be applied to obtain a very compact and high-quality wavefunction by comparing the results obtained from SHDMC with results obtained from selected CI with and without re-normalized second-order perturbation (rPT2) theory corrections. Additionally, we provide evidence that SHDMC is a robust method that can achieve convergence of both the energy and wavefunction, for various initial trial wavefunctions that differ significantly in quality. We also assess the rate of convergence of SHDMC toward the ground state and show that the method is very statistically efficient since it exhibits exponential (or near-exponential) convergence with respect to the total sampling effort. Furthermore, we explore a method to extrapolate SHDMC energies to the infinite basis limit and find that this extrapolation procedure achieves results that are within error bars of the highly accurate sCI + rPT2 extrapolation. By demonstrating the efficacy of SHDMC for this test case, we anticipate that the further validation and testing of SHDMC in applications to strongly correlated and other complex 2D materials, is within practical reach.

The remainder of the manuscript is organized as follows: In the theory section some background information on fixed-node DMC is presented. The general details of the iterative Self-Healing Diffusion Monte Carlo algorithm are also presented here, along with the computational details relevant to the results presented in the Results section. In the results section we demonstrate that the iterative SHDMC procedure achieves convergence of both the total energy and the wavefunction, of materials, regardless of the initial trial. We also demonstrate that the iterative SHDMC procedure can achieve an exponential rate of convergence in the energy with respect to cumulative sampling effort. Additionally, we compare results obtained from SHDMC (with both the locality approximation and T-moves^38^) with results obtained from truncated CI and selected CI. Additionally, we make comparisons with the energy obtained from extrapolating to the complete basis set limit using the sCI results with the results obtained from SHDMC. We present a DMC extrapolation scheme, applied within the context of SHDMC. This DMC based extrapolation scheme is analogous to the complete basis set extrapolation scheme, which is typically used along with CI or sCI methods. We also assess the impact of the choice of method (locality approximation or T-moves) used to account for the use of the non-local pseudopotential on the fixed-node DMC energy obtained from the SHDMC procedure. In the conclusions section we summarize the key conclusions drawn from the results.

## Methods

### Fixed-node DMC: background

Diffusion Monte Carlo enables the extraction of the ground-state wave function of a system by propagating an initially chosen distribution in imaginary time by way of stochastic simulation. The importance sampling DMC algorithm is based on the following equation, which is obtained by reformulating the imaginary-time Schrödinger equation as a diffusion equation for the distribution function, $f(\mathbf{R},\tau )$^13,39,40^:

1 $$\begin{aligned} -\frac{df(\mathbf{R},\tau )}{d \tau }&= -\frac{1}{2} \nabla ^{2} f(\mathbf{R},\tau ) + \nabla \left[ v(\mathbf{R})f(\mathbf{R},\tau )\right] \end{aligned}$$

2 $$\begin{aligned}&+ \left[ E_L(\mathbf{R}) - E_{T} \right] f(\mathbf{R},\tau ) \end{aligned}$$

Note that the right-hand side of Eq. (2) is composed of a diffusion term (first term), a drift term (second term, involving the drift velocity $v(\mathbf{R})=\Psi _\textrm{T}^{-1}\nabla \Psi _\textrm{T}$), and a branching term (third term, involving the local energy $E_L(R)=\Psi _\textrm{T}^{-1}\mathcal {H}\Psi _\textrm{T}$)^41^. The solution to Eq. (2) is the distribution function, $f(\mathbf{R},\tau )$, which is defined as follows.

3 $$\begin{aligned} f(\mathbf{R},\tau )=\Psi _{\textrm{T}}(\mathbf{R}) \Psi (\mathbf{R},\tau ) \end{aligned}$$

$\Psi _{\textrm{T}}$ is a trial wavefunction and $\Psi (\mathbf{R},\tau )$ is a solution of the imaginary-time Schrödinger equation. Note that by starting from some initial distribution function, the solution to Eq. (2) can be simulated by an ensemble of random walkers^42^. At the start of an importance sampling DMC calculation $(\tau =0)$ an ensemble of initial configurations is typically generated according to the probability density of the chosen trial wavefunction (Eq. 4)^13^.

4 $$\begin{aligned} f(\mathbf{R},\tau =0)= \Psi _{\textrm{T}}(\mathbf{R})\Psi _{\textrm{T}}(\mathbf{R}) \end{aligned}$$

At a given time, $\tau +\Delta \tau$, the distribution function, $f(\mathbf {R'},\tau +\Delta \tau )$, is given by the following integral equation.

5 $$\begin{aligned} f(\mathbf {R'},\tau +\Delta \tau ) = \int d\mathbf{R} \tilde{G}(\mathbf {R'},\mathbf{R},\Delta \tau )f(\mathbf{R},\tau ) \end{aligned}$$

In Eq. (5), $\tilde{G}(\mathbf {R'},\mathbf{R},\Delta \tau )$ is approximated in practice by a product of short-time Green’s functions. In practice, the action of $\tilde{G}(\mathbf {R'},\mathbf{R},\Delta \tau )$ on $f(\mathbf{R},\tau )$ is realized as a weighted random walk with the use of an acceptance/rejection step during which the proposed move from $\mathbf{R}$ to $\mathbf {R'}$ is either accepted or rejected (Metropolis-Hastings algorithm^42^. $\tilde{G}(\mathbf {R'},\mathbf{R},\Delta \tau )$ contains a drift, a diffusion, and a branching component. The drift component ensures that the random walk is directed towards where the magnitude of the trial wavefunction is large and the diffusion component enables the random sampling of proposed moves. The branching component controls the population of walkers and is used to update the weights of the walkers^18,42,43^. In the limit of infinite time $f(\mathbf{R},\tau )$ becomes proportional to the stationary target distribution:

6 $$\begin{aligned} f(\mathbf{R})&= \lim _{\tau \rightarrow \infty } f(\mathbf{R},\tau ) \approx \Psi _{\textrm{T}}(\mathbf{R}) \Psi _{0}(\mathbf{R}) \end{aligned}$$

7 $$\begin{aligned}&= \lim _{N_{c}\rightarrow \infty } \frac{1}{N_{c}} \sum _{i=1}^{N_{c}} \mathcal {W}_{i} \delta (\mathbf{R}-\mathbf{R}_{i}) \end{aligned}$$

In Eq. (7) $\Psi _{0}(\mathbf{R})$ is the lowest energy wavefunction that shares the nodes of $\Psi _{\textrm{T}}$. $\mathcal {W}_{i}$ is the statistical weight for walker *i* with the configuration $\mathbf{R}_{i}$. During the course of a DMC calculation $f(\mathbf{R},\tau )$ is updated iteratively until a sufficiently large number of configurations are sampled and the stationary distribution is realized^13,40^. Once the stationary distribution $f(\mathbf{R})$ is realized the ground state energy can be obtained as follows:

8 $$\begin{aligned} \langle E_{0} \rangle = \frac{\int f(\mathbf{R}) \left[ \frac{\mathcal {H}\Psi _{\textrm{T}}}{\Psi _{\textrm{T}}} \right] d\mathbf{R}}{\int f(\mathbf{R}) d\mathbf{R}} \approx \frac{\sum _{i=1}^{N_{c}} \mathcal {W}_{i} E_{L}(\mathbf{R}_{i})}{\sum _{i=1}^{N_{c}} \mathcal {W}_{i}} \end{aligned}$$

In Eq. (8), $\mathcal {{W}}_{i}$ is the weight for walker *i* in the configuration $\mathbf{R}_{i}$ and $E_{L}(\mathbf{R}_{i})$ is the corresponding local energy^13,39^. Similarly, the (mixed) expectation value of a local observable in $\mathbf{R}$ is obtained in FN-DMC as follows:

9 $$\begin{aligned} \langle A \rangle&= \frac{\langle |{\Psi _\textrm{T}}|{\hat{A}}|{\Psi _0}\rangle }{{\langle \Psi _\textrm{T}}|{\Psi _0}\rangle } = \frac{\int d\mathbf{R} f(\mathbf{R}) \Psi _\textrm{T}^{-1}A\Psi _\textrm{T}}{\int d\mathbf{R}f(\mathbf{R})} \end{aligned}$$

10 $$\begin{aligned}&\approx \frac{\sum _{i=1}^{N_{c}} \mathcal {W}_{i} A(\mathbf{R}_{i})}{\sum _{i=1}^{N_{c}} \mathcal {W}_{i}} \end{aligned}$$

Without the importance sampling procedure presented above, an unrestricted DMC approach will in general converge to the lowest energy state, a bosonic ground state wavefunction, which reflects the fermion sign problem. The most commonly utilized method to handle the fermion sign problem in continuum DMC is the fixed-node approximation^14,15^. The fixed-node approximation works by using the nodal surface of a trial wavefunction, $\Psi _{\textrm{T}}$, to enforce the nodal surface of the projected ground-state wavefunction. This is done by partitioning the sample space into sub-volumes that are bounded by the nodes of $\Psi _{\textrm{T}}$ and then solving Eq. (2) in each of these bounded sub-volumes or “nodal pockets”. In each nodal pocket or sub-volume $(\upsilon ^{\alpha })$ a fixed-node ground-state wavefunction ($\Psi _{\textrm{FN}}^{\alpha }$) and a corresponding fixed-node ground-state energy $(E_{FN}^{\alpha })$ is obtained^44^.

In practice, the fixed-node approximation is realized by rejecting any moves or killing the walkers that cross a node of the trial wavefunction.

A key aspect of the fixed-node approximation is that, unless the nodal surface of the trial wavefunction is identical to that of the true fermionic ground-state wavefunction, the fixed-node DMC energy will be greater than the true ground-state energy (the variational principle). If the trial wavefunction has the exact same nodal surface as the true ground-state wavefunction, the ground-state wavefunction found for each nodal pocket will have the same energy^13^. In general, a wavefunction with the exact nodal structure is not known and must be approximated. Typically, the nodal structure of trial wavefunctions is optimized indirectly via *e.g.* a VMC energy minimization procedure. In this work, we assess the performance of an approach that directly uses DMC imaginary time projection as the vehicle to improve the nodal structure of the trial wavefunction.

### Self-healing diffusion Monte Carlo

The main observation in Self-Healing Diffusion Monte Carlo (SHDMC) is that the constrained fixed-node projection operation contains sufficient information to systematically improve the trial wavefunction in an iterative manner. For imperfect nodes the fixed node ground state wavefunction $\Psi _0$ must have a discontinuity of the gradient (a kink) at the node^45^. Locally smoothing away the kink of $\Phi _0$ by a convolution with smooth approximation of the delta function $\tilde{\delta }(\mathbf{R})$, improves the nodes^35,36^.

In SHDMC we consider a class of trial wavefunctions of the form

11 $$\begin{aligned} \Psi _\textrm{T}(\mathbf{R}) = \sum _n c_n e^{J_\textrm{T}(\mathbf{R})}\Phi _n(\mathbf{R}) \end{aligned}$$

where $J_T(R)$ is a given trial Jastrow factor and the functions $\Phi _n$ comprise an orthonormal basis, *i.e.* $\langle {\Phi _n}|{\Phi _m}\rangle =\delta _{nm}$. The functions $\Phi _n$ and their gradients must be continuous so a truncated expansion of the delta function results in a smoothing operator.

A better approximation to $\Psi _T$ can be obtained directly from $\Psi _0$ if we can compute:

12 $$\begin{aligned} \Psi _T(\mathbf{R}) = \int \mathbf{dR^\prime } \tilde{\delta }{(\mathbf{R}^\prime ,\mathbf{R})} \Psi _0(\mathbf{R}^\prime ). \end{aligned}$$

SHDMC uses approximations to the delta function of the form

13 $$\begin{aligned} \tilde{\delta }= \sum _n^* e^{J_T} |\Phi _n \rangle \langle \Phi _n | e^{-J_T}, \end{aligned}$$

which leads to new coefficients as follows:

14 $$\begin{aligned} c_n = \langle \Phi _n |{e^{-J_\textrm{T}}} |{\Psi _0} \rangle \end{aligned}$$

The $*$ in Eq. (13) indicates that only a finite number of coefficients $(c_n)$ are included in the sum (see 2.2). Because the sum in Eq. (13) is finite and the functions $\Phi _n$ and their gradients are continuous at the node Eq. (12), when sampled in DMC with sufficient statistics, yields a trial wavefunction with better nodes^35,36^.

Up to a normalization constant, the updated coefficients $c_n$ can be estimated during a DMC simulation as follows:

15 $$\begin{aligned} c_n&\propto \frac{\langle {\Psi _\textrm{T}}|{\frac{\Phi _n^*}{\Psi _\textrm{T}^*}e^{-J_\textrm{T}}}|{\Psi _0}\rangle }{\langle {\Psi _\textrm{T}}|{\Psi _0}\rangle } = \frac{\int d\mathbf{R} f(\mathbf{R}) \frac{\Phi _n^*(\mathbf{R})}{\Psi _\textrm{T}^*(\mathbf{R})}e^{-J_\textrm{T}(\mathbf{R})}}{\int d\mathbf{R}f(\mathbf{R})} \end{aligned}$$

16 $$\begin{aligned}&\approx \frac{\sum _{m=1}^{N_c} \mathcal {W}_m \frac{\Phi _n^*(\mathbf{R}_m)}{\Psi _\textrm{T}^*(\mathbf{R}_m)}e^{-J_\textrm{T}(\mathbf{R}_m)}}{\sum _{m=1}^{N_c} \mathcal {W}_m} \end{aligned}$$

Here, *f*(*R*) represents the mixed distribution, which when sampled in DMC as a collection of $N_c$ walker configurations at points $R_m$ is $f(R)=\Psi _T(R)\Psi _0(R)=\sum _{m=1}^{N_c}\mathcal {W}_m\delta (R,R_m)$.

#### The SHDMC algorithm

The SHDMC algorithm consists of performing a sequence of fixed-node DMC calculations where the coefficients estimated in the current iteration are used to construct the trial wavefunction entering the following one^35,36^.

Since the estimated coefficients have attendant statistical uncertainty, a coefficient filtering and averaging scheme is applied based on the estimated noise. We next describe the details of this approach.

During a given iteration of SHDMC a standard DMC run is performed. After equilibration, each coefficient is evaluated for the $N_c$ walker configurations and averages, $\langle c_n \rangle$, and their associated standard errors $\left( \frac{\kappa _n^{1/2}\sigma _{c_n}}{N_c^{1/2}} \right)$ are subsequently calculated. In order to avoid underestimating $\frac{\sigma _{c_{n}}}{N_{c}^{1/2}}$ a re-blocking scheme^46^ is used to estimate the autocorrelation time,$\kappa _n$, for each coefficient. After obtaining the estimates of the averages and standard errors of the coefficients, a magnitude/error based cutoff criteria is applied to filter the coefficients. This filtering step removes coefficients with excessive noise, which helps prevent moving the nodes of the newly obtained fixed-node wavefunction in completely arbitrary directions. Therefore, the sum in Eq. (13) is restricted according to the specific cutoff criteria defined below (Eq. 17).

17 $$\begin{aligned} \big | \langle c_{n} \rangle \big | \ge 4 \frac{\sigma _{c_{n}}}{N_{c}^{1/2}} \end{aligned}$$

The application of this cutoff criteria (statistical significance at the “4-sigma” level) determines which coefficients will be allowed to have a nonzero magnitude at the start of the next iteration of SHDMC.

It is relevant to note that the application of this cutoff criteria does not prevent coefficients that failed to satisfy Eq. (17) from being sampled during the DMC calculation step of the next iteration. Instead, the application of this cutoff criteria enables the re-estimation of previously unresolved coefficients, while limiting the occurrence of arbitrary changes to the nodes of the iteratively updated trial wavefunction.

During the course of the SHDMC procedure, fluctuations in the fixed-node wavefunction obtained from successive iterations are monitored. The presence of significant fluctuations indicates that an increase in sampling effort is warranted in order to prevent statistical noise from making an unfavorable impact on the quality of the wavefunction and to facilitate convergence of the SHDMC procedure. The criteria used to detect significant fluctuations in the wavefunction between successive iterations is defined below.

18 $$\begin{aligned} \langle \mathbf{C}_{i} - \mathbf{C}_{i-1} \rangle \times \langle \mathbf{C}_{i-1} - \mathbf{C}_{i-2} \rangle \le 0 \end{aligned}$$

In Eq. (18) $\mathbf{C}_{i}$, $\mathbf{C}_{i-1}$, and $\mathbf{C}_{i-2}$ are vectors containing the coefficients of the fixed-node wavefunctions obtained from iterations *i* (current iteration), $i-1$, and $i-2$, respectively. When this criteria is met the sampling effort to be used during the DMC step of the next iteration is increased by multiplying the number of samples used during the current iteration (iteration *i*) by a fixed scale factor (we used a scale factor of 1.4 in this work). Incorporating both the cutoff criteria (Eq. 17) and the criteria defined in Eq. (18) in the SHDMC algorithm helps move the nodes of an initial fixed-node trial wavefunction towards the nodes of the true ground-state wavefunction in a controlled systematic manner and facilitates convergence of the wavefunction^35,36^. It is relevant to note that SHDMC does not require the use of any particular basis set family. If desired, SHDMC calculations can be performed using alternative bases, such as plane waves and splines, or a basis constructed using Gaussian type orbitals. This makes SHDMC transferable to other classes of systems and materials which require the use of different bases. The only requirements of the basis are that the orbitals must be orthogonal and the cutoff applied to the many-body wavefunctions must be energy based, so that it approaches a delta function like Gaussian. The functions $\Phi _n$ and their gradients must be continuous so a truncated expansion of the delta function results in a smoothing operator.

### Computational details

An initial single-reference wavefunction for monolayer graphene was obtained within the PBE approximation to DFT with PySCF^47,48^. The atomic core of carbon was represented by a the corresponding ccECP and a cc-pVQZ atomic basis was used in the DFT calculations^49^. Subsequently, a selected CI calculation (Configuration Interaction Perturbatively Selected Iterative, or CIPSI method^50^ was used to generate a large 18 million determinant wavefunction for the primitive cell at the gamma point. The active space for subsequent SHDMC calculations was constructed by excluding determinants with squared coefficients falling below a cutoff value.

The VMC and DMC calculations in this work were performed using QMCPACK^43,51^. The equations presented in section 1, necessary for performing SHDMC calculations, were also implemented in QMCPACK.

For each iteration of the SHDMC procedure, an initial VMC calculation was performed, followed by a DMC calculation with a time step of $(\Delta \tau =0.02~\textrm{Ha}^{-1})$, consisting of 2000 sequential MC steps. For the main DMC calculation a time step of $\Delta \tau =0.01~\textrm{Ha}^{-1}$ was employed. During the SHDMC procedure, the sampling effort is increased according to the criteria described in Eq. (18) by increasing the number of MC steps prescribed for the main DMC calculation. For the first DMC calculation used to initiate the SHDMC procedure a total of 25.6 million configurations were sampled. The total number of configurations sampled is calculated as $N_c=\textrm{N}_{\textrm{blocks}} \times \textrm{N}_{\textrm{steps}} \times \textrm{N}_{\textrm{walkers}} = 2000 \times 25 \times 512$. For all of the subsequent SHDMC iterations, a consistent target population of 512 walkers was maintained.

All of the SHDMC calculations reported in the results section were performed with the statistical parameters described above. Unless noted otherwise all of the SHDMC calculations were performed within the locality approximation^52,53^ to handle non-local pseudopotentials. For the purpose of comparison, all the SHDMC calculations reported below were performed using the same Jastrow factor. The Jastrow was optimized in VMC for the single determinant constructed from PBE Bloch orbitals.

#### Computational cost

All SHDMC calculations were performed using a 13 node cluster with 128 CPUs per node. The vast majority of the SHDMC iterations were performed using a single node and the most computationally demanding iteration was performed using all 13 of the nodes. On a relatively small cluster, including human-supervision time, it took approximately 3 months to obtain all of the results presented in this work. To achieve convergence of the SHDMC iterative procedure it took $\sim 10^{5}$ CPU hours on this machine.

sCI via the CIPSI method was used to produce reference/benchmark data for comparison with SHDMC. sCI multideterminant wavefunctions were constructed from PBE orbitals obtained from PySCF in cc-pVDZ, cc-pVTZ, cc-pVQZ, cc-pV5Z, and cc-pV6Z atomic basis sets. To ensure the inclusion of important basis functions with small exponents, the traditional exponent cutoff of 0.1 was reduced to 0.08 for all basis sets. This adjustment was particularly important for the larger cc-pV5Z and cc-pV6Z sets, as the 0.1 cutoff led to inconsistent energies, whereas the smaller basis sets were unaffected by this change.

The sCI calculations were refined by performing a single round of natural orbital (NO) transformations from PBE orbitals after initially converging the wavefunctions in a first round of CIPSI calculations. The iterative procedure of adding selected determinants was stopped once the rPT2 correction fell below 0.5 mHa for cc-pVDZ, cc-pVTZ, and cc-pVQZ, and below 2mHa for cc-pV5Z and cc-pV6Z, leading to an initial number of determinants ranging from 1 million to 34 million, depending on the basis set. This process resulted in a significant reduction in the size of the multideterminant expansion, with a PT2 value equal to or below 0.5 mHa for all basis sets and 1mHa for cc-pV6Z.

The resulting variational energies ($E_{var}$) and perturbatively corrected energies ($E_{var}$ + rPT2) were extrapolated to an infinite determinant count as the rPT2 correction approached zero (see Supplementary Information). These rPT2-extrapolated values for each basis set were subsequently used to extrapolate to the complete basis set (CBS) limit using a three-point formula.

**Fig. 1:**
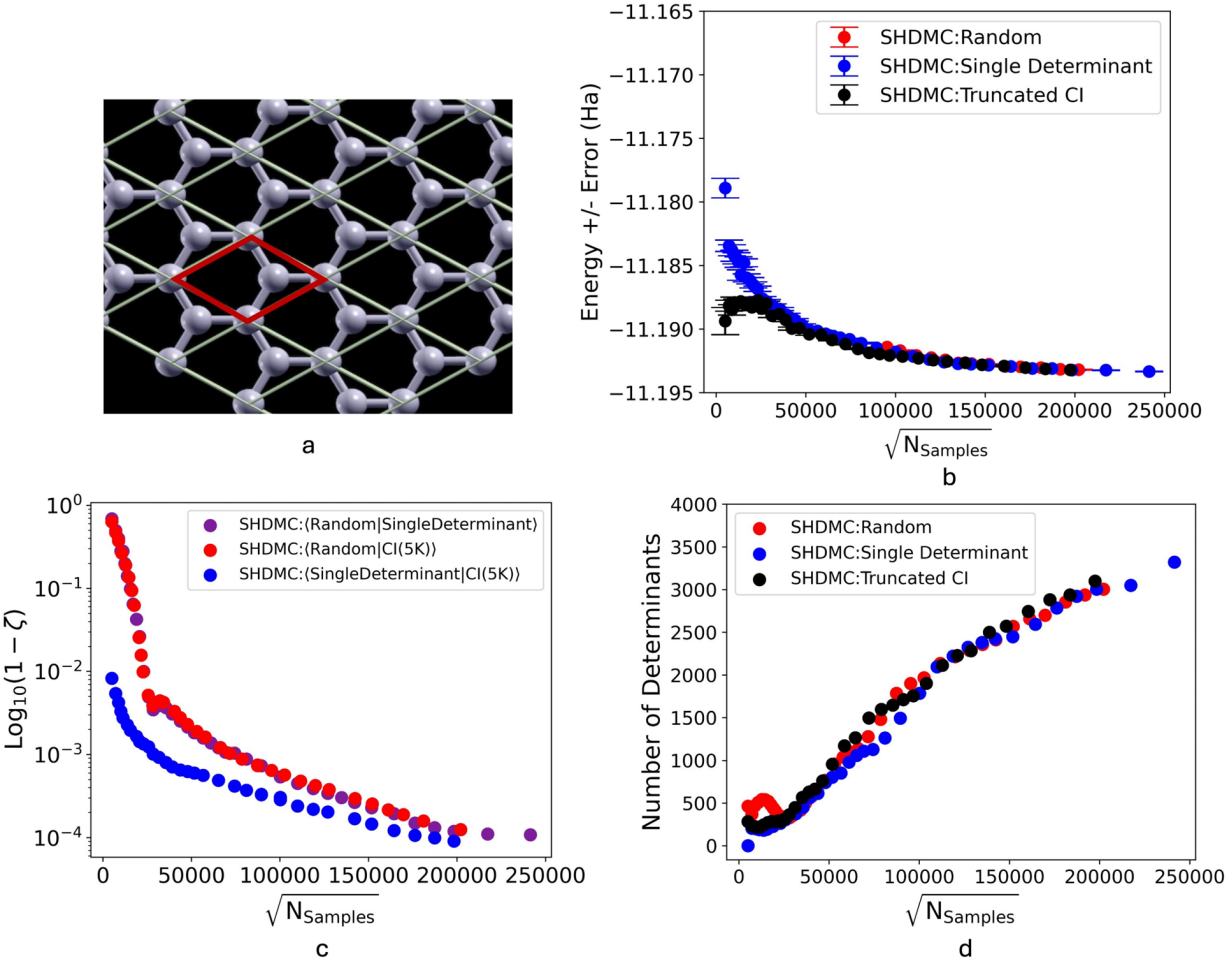
Analysis of SHDMC results in 5000 determinant active space. Figure 1a is a depiction of the graphene monolayer. In 1b-1d, various quantities are shown with respect to the square root of the cumulative number of samples collected through all SHDMC iterations, $\sqrt{\mathrm {N_{samples}}}$, which represents the total computational cost of SHDMC. Figure 1b demonstrates convergence of the SHDMC total energy, independent of the initial trial wavefunction. Trajectories for random, single determinant, and truncated CI initial trial wavefunctions are shown in red, blue, and black, respectively. The error bars indicate the error in the Monte Carlo estimate of the the energy (error in the DMC energy). Figure 1c indicates the difference from unity in the determinantal overlap between pairs wavefunctions produced along the same SHDMC trajectories. Figure 1d indicates the number of determinants retained in the SHDMC statistical selection process along each trajectory. For all initial trial wavefunctions a similar number of determinants are retained once a cumulative sampling effort of $\sqrt{\mathrm {N_{samples}}} \approx 25,000$ is reached. The SHDMC active space is comprised of 5000 determinants in each case shown here.

## Results and discussion

In this section we present evidence that Self-Healing Diffusion Monte Carlo can be applied to obtain a high-quality and very compact wavefunction for graphene (single unit cell at the $\Gamma$ point) as an example 2D material (see Figure 1a). While calculations in systems with larger atomic counts are possible with SHDMC^38^, performing SHDMC on a single unit cell enables us to benchmark SHDMC results against selected CI results, including the results obtained from extrapolating to the complete basis set limit. Additionally, we provide evidence that SHDMC is capable of achieving convergence of both the total energy and the wavefunction of this system irrespective of the initially chosen coefficients of the trial wavefunction. We also assess the rate of convergence of the iterative SHDMC procedure. We compare SHDMC (with both the locality approximation and T-moves^38^) with truncated CI and selected CI. We also make comparisons with the energy obtained from extrapolating to the complete basis set limit using the sCI results. In this section we also propose a scheme for extrapolating SHDMC results to the infinite determinant limit and compare it with the selected CI complete basis set extrapolation. It is relevant to note that all of the SHDMC calculations were done using the cc-pVQZ basis set.

### Energy and wavefunction convergence of the iterative SHDMC algorithm

For the purpose of investigating the convergence properties of the SHDMC algorithm with respect to variations in the initial trial wavefunction, we performed three independent SHDMC runs starting from initial trial wavefunction of differing quality (poor, standard, and high-quality). The poor-quality initial trial wavefunction (“Random”) was generated by assigning the coefficients of the first five determinants of the multi-determinant expansion to be uniform random numbers between -0.1 and 0.1 and the remaining coefficients were assigned to be equal to zero. The standard-quality wavefunction (“Single Determinant”) was generated by assigning the coefficient of the first determinant of the multi-determinant expansion (the PBE ground state) to be equal to one and the remaining coefficients were set equal to zero. The high-quality wavefunction was generated by truncating a multi-determinant wavefunction, obtained using selected CI (“Truncated CI”). The active space considered for SHDMC for all three of these initial trial wavefunctions was comprised of five-thousand determinants and all trial wavefunctions were normalized before beginning the SHDMC calculations.

In Figure 1b we see that the total energy of graphene (single 2-atom unit cell), obtained using the iterative SHDMC procedure, converges to the same value (sub-mHa scale) for different initial trial wavefunctions. For the purpose of effectively displaying the data, the total energies obtained from the first 15 iterations of the SHDMC procedure, when starting from the random initial trial wavefunction, were excluded from Figure 1b.

The total energy of graphene (single unit cell) obtained from the iterative SHDMC procedure, when starting from the random initial trial wavefunction, was found to be $-11.19320(4)$ Ha. The converged total energies obtained when starting from the single determinant initial trial wavefunction and the truncated CI initial trial wavefunction are $-11.19332(2)$ Ha and $-11.19319(3)$ Ha, respectively. The value of $\sqrt{N_{\textrm{samples}}}$ at this level of convergence was $2.02\times 10^{5}$, 2.41$\times 10^{5}$, and 1.97$\times 10^{5}$ when starting from the random, single determinant, and truncated CI initial trial wavefunctions, respectively. As expected, the cumulative computational cost required to achieve convergence is higher when starting from the poor-quality initial trial wavefunction (random). Although the cumulative computational cost required to achieve convergence is comparatively high when starting from the random initial trial wavefunction, it is not prohibitively high and convergence is still achieved.

In Figure 1c we present results regarding the convergence of SHDMC at the level of the wavefunction coefficients, rather than the total energy. By assessing the coefficient overlap between different SHDMC trajectories, we show that the SHDMC algorithm eventually finds the same wavefunction regardless of the initially chosen coefficients of a multideterminant trial wavefunction.

**Fig. 2:**
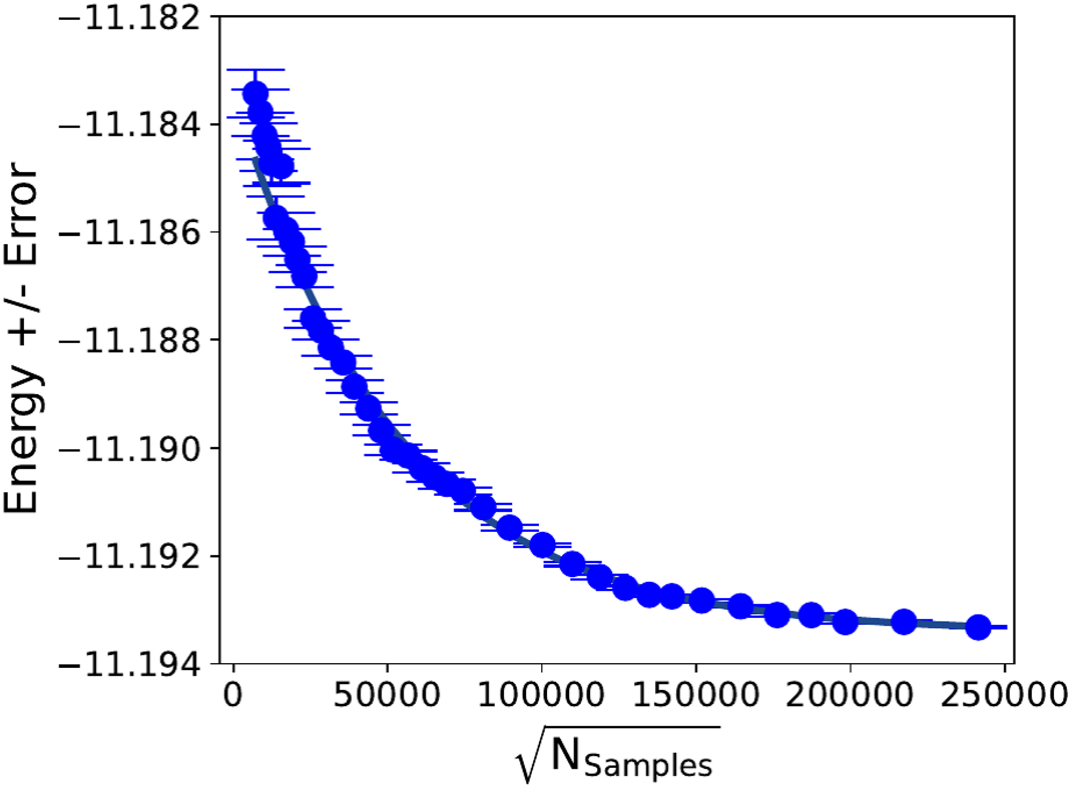
Energy convergence of SHDMC iterative algorithm. Energy vs. square root of cumulative computational cost (single determinant initial trial wavefunction) fit to the decaying exponential $E = \Delta E e^{\sqrt{N_{\textrm{samples}}}/r}+E_0$, with $\Delta E=10~\textrm{mHa}$, $r=5.26\times 10^4$, and $E_0=-11.193~\textrm{Ha}$. The variance weighted coefficient of determination of the fit was $R^{2}=0.936$.

In Figure 1c, $\zeta$ represents the overlap between the anti-symmetric (multi-Slater determinant) component of the SHDMC wavefunctions obtained when starting from the different initial trial wavefunctions. Formally, $\zeta$ is calculated as shown below.

19 $$\begin{aligned} \zeta = \frac{\langle C^{X} \mid C^{Y} \rangle }{\parallel C^{X} \parallel \parallel C^{Y} \parallel } \end{aligned}$$

In Eq. (19), $C^{X}$ and $C^{Y}$ are the vectors containing the coefficients of the SHDMC wavefunctions, obtained when starting from any combination of the different initial trial wavefunctions. Corresponding pairs of $C^{X}$ and $C^{Y}$ obtained from iterations with the most similar costs were used to calculate each $\zeta$. By examining Figure 1c it can be observed that over the course of the iterative SHDMC process the gradually improving wavefunctions, obtained when starting from the different initial trial wavefunctions, become more and more similar. In Figure 1d we see that once a cumulative sampling effort of $\sqrt{\mathrm {N_{samples}}} \approx 25,000$ is reached a similar number of determinants are retained during the course of the SHDMC iterative procedure, for each of the different initial trial wavefunctions.

**Fig. 3:**
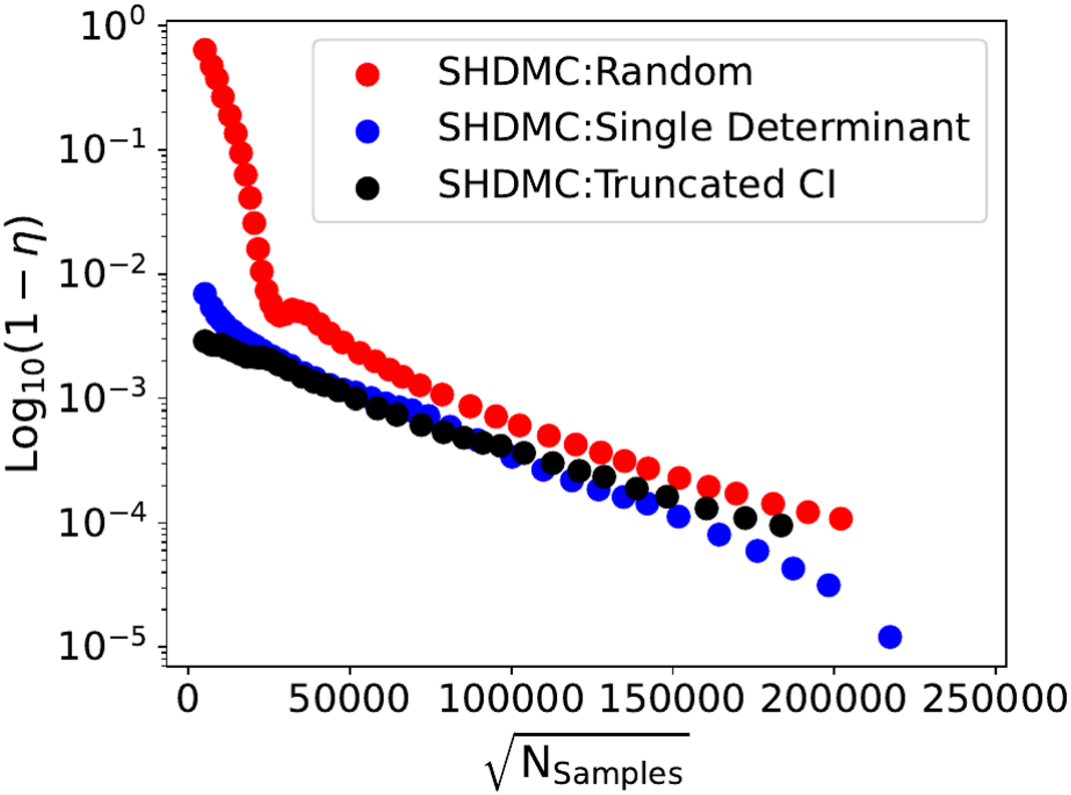
Wavefunction convergence of SHDMC iterative algorithm. Measurement of overlap between wavefunctions from each iteration of SHDMC obtained when starting from the random initial trial wavefunction (SHDMC:Random), the single determinant initial trial wavefunction (SHDMC:Single Determinant), and the truncated CI(5K) initial trial wavefunction (SHDMC:Truncated CI) with the final wavefunction obtained when starting from the Single Determinant initial trial wavefunction.

### Rate of convergence of the iterative SHDMC algorithm

In Figure 2 the curve obtained by plotting total energy as a function of the square root of the cumulative computational cost, when starting from the single determinant initial trial wavefunction, is accurately modeled by a decaying exponential function. This is similar to what has been previously observed regarding the rate of convergence of the iterative SHDMC procedure in model systems^35^.

**Fig. 4:**
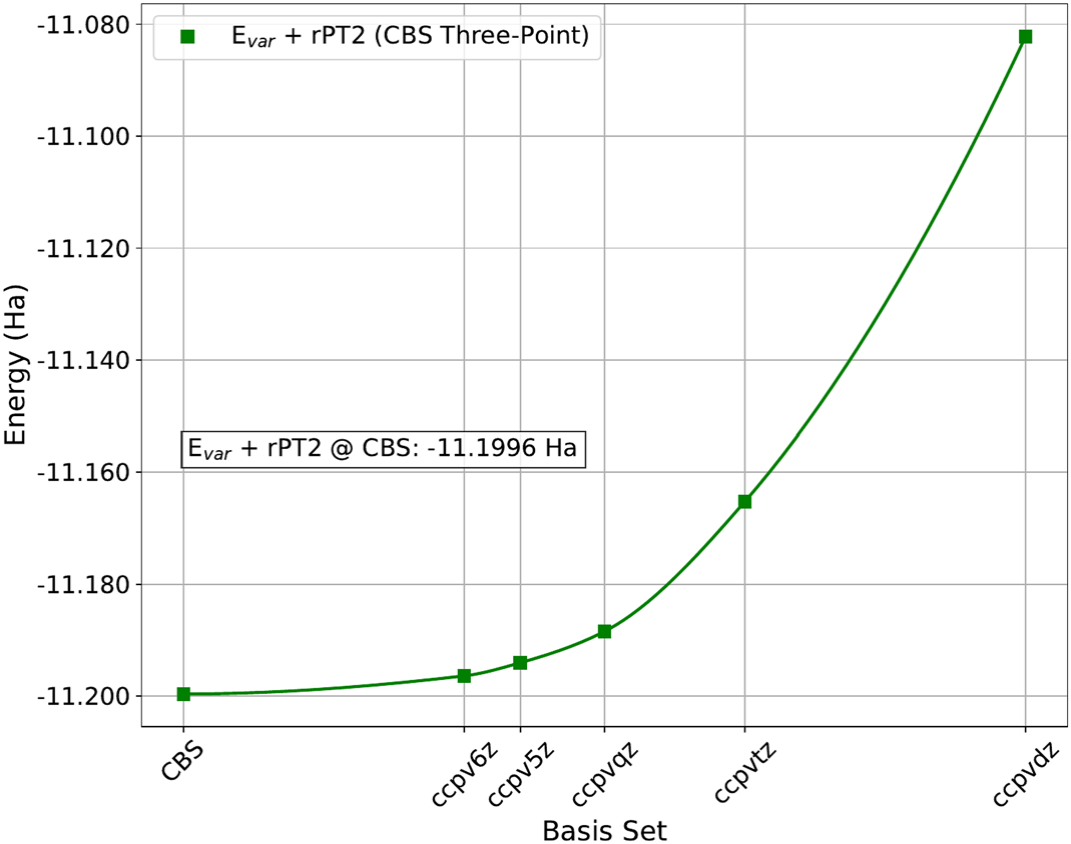
Selected CI Complete Basis Extrapolation. Converged sCI total energies in each basis, extrapolated to the complete basis (CBS) limit with a three-point formula^54^. The sCI energies, variational ($E_{var}$) and variational plus rPT2 corrections ($E_{var}$+rPT2), were first extrapolated to infinite determinant count limit ($|\textrm{rPT2}|\rightarrow 0$ limit) as described in the main text. The line is a guide to the eye.

In Figure 3 we provide a demonstration of how the wavefunction changes during the course of the SHDMC iterative procedure when starting from the three different initial trial wavefunctions. In Figure 3, $\eta$ represents the overlap between the wavefunctions obtained from consecutive iterations of SHDMC when starting from the different initial trial wavefunctions. Formally, $\eta$ is calculated as shown below.

20 $$\begin{aligned} \eta = \frac{\langle C^{N} \mid C^{Max} \rangle }{\parallel C^{N} \parallel \parallel C^{Max} \parallel } \end{aligned}$$

In Eq. (20), $C^{N}$ represents the vectors containing the coefficients of the SHDMC wavefunctions obtained from iterations *N* and $C^{Max}$ is the vector containing the coefficients of the wavefunction used for the final iteration of the SHDMC procedure, when starting from the single-determinant initial trial wavefunction. By examining Figure 3 we can see that, when starting from the random initial trial wavefunction, the difference in the wavefunctions obtained from early iterations are drastically different than the final SHDMC:Single Determinant wavefunction. This is expected when using a poor-quality intitial trial wavefunction. The quantity $1-\eta$ provides a metric by which convergence of the wavefunction can be assessed. When convergence of the wavefunction is achieved $1-\eta$ approaches zero.

**Fig. 5:**
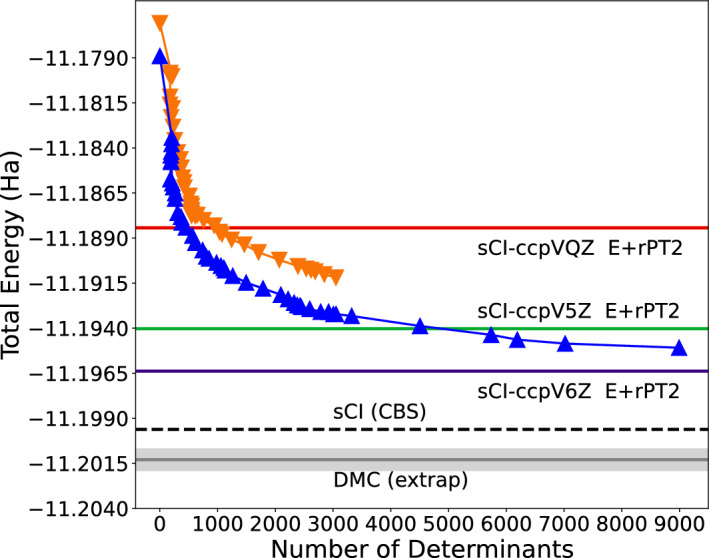
Comparison of total energies obtained with selected CI and SHDMC. Total energies obtained from each iteration of SHDMC (QZ basis) are shown with orange and blue triangles for T-moves and the locality approximation, respectively. Selected CI rPT2 corrected energies for different atomic basis sets are shown as solid horizontal lines. The CBS extrapolated selected CI energy (− 11.1996 Ha) is shown as a dashed black line, while the determinant extrapolated DMC value we obtain is shown in grey.

### Comparison between SHDMC and selected CI

The converged sCI + rPT2 and selected CI Complete Basis Extrapolation results are displayed in Figure 4. In Table 1, we compare converged SHDMC results in an active space composed of 20,000 determinants (cc-pVQZ only) and sCI total energies obtained for graphene, along with the rPT2 corrections to the sCI energy. Additionally, the number of determinants composing the converged SHDMC and sCI wavefunctions are presented. The number of determinants enables a direct analysis of the compactness of the wavefunctions obtained from the aforementioned methods. Therefore, we place emphasis on both the total energy and compactness as indicators of the quality and utility of these wavefunctions.

**Table 1:** Comparison between SHDMC (20K determinant active space) and Selected CI.

Method	Total Energy (Ha)	Abs. Error (mHa)	No. Dets
sCI-NO-5Z $E_\textrm{var}$	-11.193464	6.2(1)	$14.6\times 10^6$
sCI-NO-5Z extrap.	-11.194027	5.6(1)	$\infty$
SHDMC-TM-QZ	-11.19446(6)	5.2(1)	8994
SHDMC-LA-QZ	-11.19481(6)	4.8(1)	8994
sCI-NO-6Z $E_\textrm{var}$	-11.194964	4.6(1)	$3.5\times 10^6$
sCI-NO-6Z extrap.	-11.196381	3.2(1)	$\infty$
sCI (CBS)	-11.1996(1)	0.0(1)	$\infty$
SHDMC extrap.	-11.2013(6)	-1.3(7)	$\infty$

Comparison between total energies (in units of Hartrees), obtained from SHDMC with T-moves (SHDMC-TM-QZ) and with the locality approximation (SHDMC-LA-QZ), sCI total energies (sCI-NO-5Z and sCI-NO-6Z), and sCI +rPT2 (sCI-NO-5Z extrap. and sCI-NO-6Z extrap.) The sCI-NO-5Z extrap. and sCI-NO-6Z extrap. energies are obtained by extrapolating to the infinite determinant limit (where the rPT2 correction to the energy approaches to zero). The SHDMC-TM-QZ and SHDMC-LA-QZ total energies are the average local energies (with statistical re-blocking) obtained in the limit of timestep = 0 $\textrm{Ha}^{-1}$, using the converged SHDMC wavefunction as the trial wavefunction. The errors in the SHDMC-TM-QZ and SHDMC-LA-QZ total energies are the statistical errors in the estimate of the average local energy (DMC total energy). The absolute errors in the total energies (in units of millihartrees) are given as the difference between any of the given total energy values and the sCI (CBS) total energy. The total energy obtained from extrapolating SHDMC results to the infinite determinant limit (SHDMC extrap.) is compared to the selected CI energy obtained from extrapolating to the complete basis set limit (sCI (CBS)). The number of determinants, with a nonzero weight, used to construct each multideterminant wavefunction is provided in the column labeled No. Dets. Both the SHDMC-TM-QZ and SHDMC-LA-QZ wavefunctions were obtained using a 20,000 determinant active space.

After the SHDMC wavefunction converged in the 5000 determinant active space a new trial wavefunction was generated by extending the wavefunction to contain 20,000 determinants. The SHDMC procedure provided updated coefficients for the determinants within the extended 20K active space with 8994 having non-zero weight, according to the criteria given by Eq. (17).

Within this active space, the total energy obtained from SHDMC, both with the locality approximation and T-moves, is lower than the sCI + rPT2 total energy obtained in the cc-pV5Z basis, but higher than the total energy obtained in cc-pV6Z basis (see Figure 5). The total energy obtained from SHDMC with the cc-pvQZ orbital basis is -11.9481 $\pm 6.1119 \times 10^{-5}$ when employing the locality approximation and -11.9446 $\pm 5.8706 \times 10^{-5}$ with t-moves. The total energy obtained from sCI + rPT2 with the cc-pvQZ basis is $6.3760 \times 10^{-3}$ Ha greater than the total energy from SHDMC with the locality approximation and $6.0281 \times 10^{-3}$ Ha greater than the total energy from SHDMC with t-moves. Exceeding the quality of DMC require a very large atomic basis in selected CI as well as an active space with $10^6-10^7$ determinants.

We additionally performed extrapolations to the SHDMC infinite determinant count limit within the QZ basis. As described above, SHDMC was first performed separately with T-moves and the locality approximation to produce two sequences of multireference wavefunctions, which we denote here as SH-TM and SH-LA, respectively. Subsequently, VMC, DMC-TM and DMC-LA calculations were performed on each of the SH-TM and SH-LA sequences with the resulting in four sets of DMC energies (DMC-TM(SH-TM), DMC-LA(SH-LA), DMC-TM(SH-LA) and DMC-LA(SH-TM) and two sets of VMC energies.

**Fig. 6:**
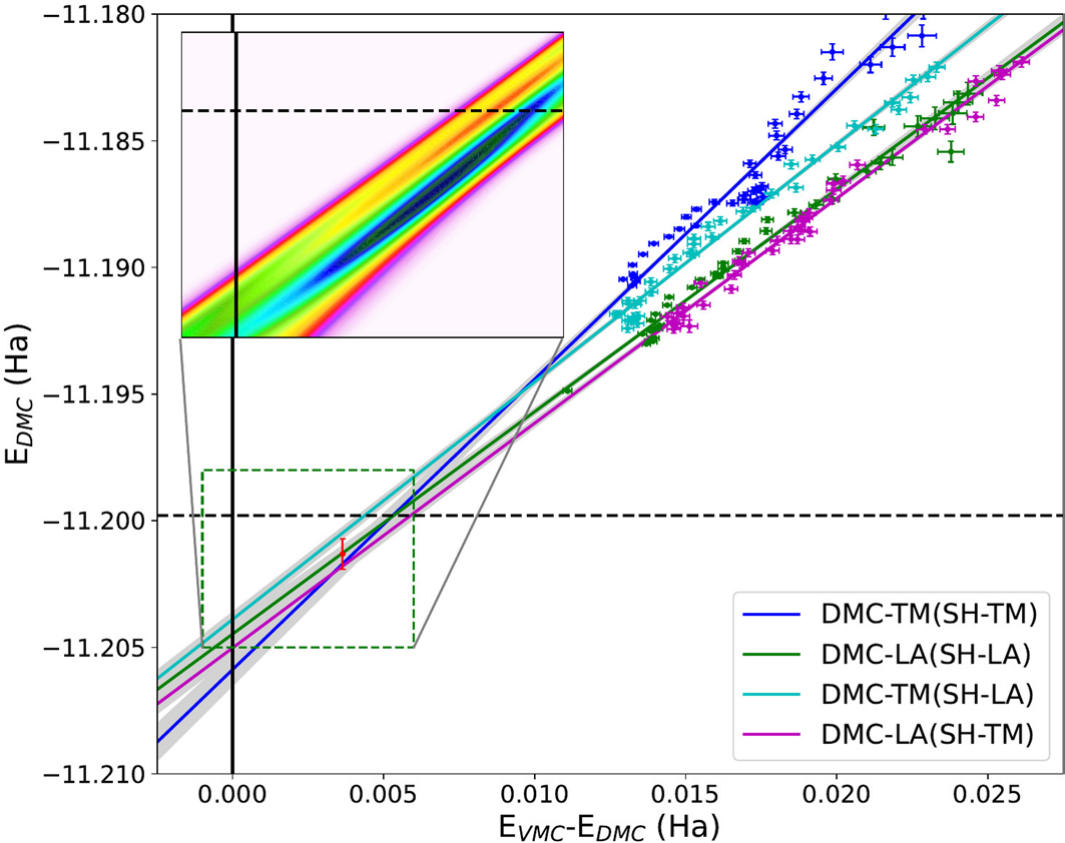
Extrapolation of DMC and VMC energies to the infinite determinant count limit in the QZ basis. SHDMC was performed separately with T-moves and the locality approximation to produce two sequences of multireference wavefunctions (SH-TM and SH-LA). VMC, DMC-TM and DMC-LA energies for each wavefunction sequence are shown and the x and y error bars represent the standard errors from Monte Carlo sampling. The black dashed horizontal line is placed at the extrapolated sCI reference value of − 11.1996 Ha. The infinite determinant count + CBS limit is reached when DMC energies cross, as indicated in the inset by a dark high probability region. The resulting DMC energy is − 11.2013(6) Ha, with error bar shown in red obtained by resampling the energy data. A finite error remains on the VMC energies due to the fixed QZ basis (no CBS extrapolation).

In Figure 6, we plot the DMC energies as a function of VMC/DMC energy difference, with the data exhibiting near linear behavior. Linear behavior in this fashion has been observed previously in other studies involving high quality wavefunctions. Using the calculated errorbars shown in the figure, we resampled the dataset (10,000 samples) and performed total least squares linear fits for each sample. The mean of each fit appears as solid colored lines on the plot, while the standard error of each fit is shown in grey. The black dashed horizontal line denotes the sCI reference value of -11.1996 Ha. In a complete basis set (larger than QZ), an extrapolation of the DMC energies to zero VMC/DMC energy difference would result in an estimate of the exact energy. However, since our VMC data retains significant basis set error, we adopt a different approach.

Since DMC operates in a complete basis within the nodal structure, DMC is quite insensitive to basis set choice and so we choose instead to locate the region where the DMC energies among the four extrapolations have the greatest agreement, thus avoiding additional error from VMC. The inset to Figure 6 shows contours of the probability distribution of the combined resampled fits of all four DMC datasets. Using maximum likelihood estimation, we locate the point of greatest agreement for DMC, shown in red in the figure. This point falls at a finite VMC/DMC energy difference, as expected in the QZ basis. At this finite VMC/DMC energy difference, we estimate the exact DMC energy as -11.2013(6) Ha, which is in close agreement with the selected CI reference value. The extrapolated point where DMC energies calculated with t-moves and the locality approximation match should be locality error free^55^. The extrapolation would be inaccurate if the multideterminant expansion cannot describe the nodal surface properly. The systematic accuracy of the extrapolation depends on the multideterminant expansion being sufficiently expressive to properly describe the nodal surface. Extrapolated approaches have been used in practice both in QMC and quantum chemistry methods. While their validity has been established in practice, we are not aware of a formal proof^56–58^. The agreement between SHDMC extrapolations and selected CI extrapolations serves as a validation of our approach.

**Fig. 7:**
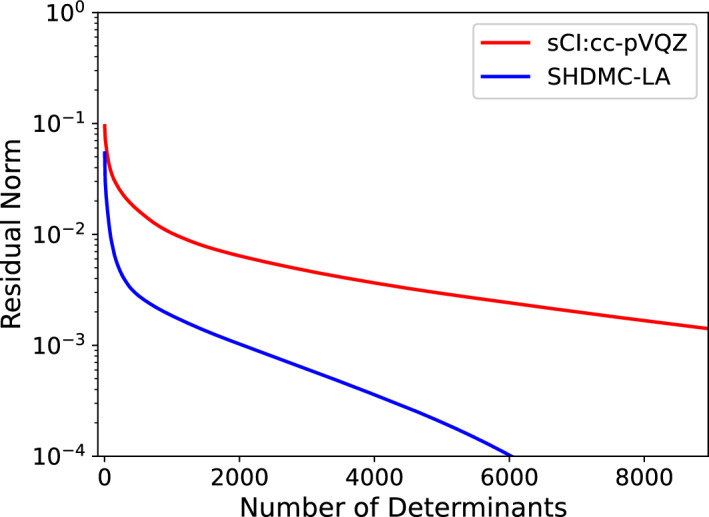
Comparison of compactness of coefficients between SHDMC and sCI in the sCI:cc-pVQZ basis. The curves labeled sCI:cc-pVQZ and SHDMC-LA demonstrate the compactness of the sCI and SHDMC-LA wavefunctions, respectively, that is achieved by truncating these wavefunctions to contain the number of determinants indicated on the abscissa. The residual norm (ordinate) provides a way to measure the compactness of the sCI and SHDMC-LA wavefunctions.

In Figure 7 we investigate the compactness of the fixed-node wavefunctions obtained from the iterative SHDMC procedure compared to the compactness of the sCI wavefunction (cc-pVQZ basis). We define the residual norm as $r_N=1-\sum _{i=1}^Nc_i^2$, where *N* ranges from one to the total number of determinants in the wavefunction. A wavefunction with more rapidly decreasing $r_N$ is more compact. In Figure 7, we compare the residual norm for the final SHDMC-LA wavefunction, obtained in the 20K active space, with the sCI wavefunction using the same orbital basis (cc-pVQZ).

By comparing the residual norm of the coefficients as function of the number of determinants, used to construct the multi-determinant wavefunctions, we can see that SHDMC finds a much more compact wavefunction than is obtained by selected CI, due to the expressive Jastrow factor obtained by DMC projection. The residual norm of each wavefunction displays exponential decay ($r_N\propto ae^{-bN}$) with respect to determinant count beyond a certain threshold, as evidenced by linear or near linear behavior on the plot. However, the residual norm of the SHDMC wavefunction has a prefactor about one order of magnitude smaller than sCI as well as a larger exponent (faster decay rate). While any method approaching the full CI limit must ultimately scale exponentially, we can see that SHDMC saturates this limit rapidly, allowing for highly accurate results to be obtained with relatively small expansions.

## Conclusions

In this work we have shown that Self-Healing Diffusion Monte Carlo is capable of finding both a high-quality and very compact wavefunction for graphene (single unit cell at the $\Gamma$ point) as an example 2D material. We demonstrated this using both the locality approximaton and T-moves, despite the differing treatment of non-local pseudopotentials between the methods. We find that the iterative SHDMC procedure converges to both the same total energy and wavefunction regardless of the coefficients of the chosen initial multideterminant trial wavefunction. This aspect in particular addresses the problems of the “fixed-node error” and “locality error” that can limit practical applications of fixed-node DMC. We have also shown that SHDMC reduces the fixed node error exponentially with respect to sampling effort, which is an efficient use of statistical information for a Monte Carlo method. This further highlights the ability of SHDMC to find a high-quality wavefunction, but extremely compact wavefunction. Since the computational cost scales linearly with the number of determinants composing the trial wavefunction, a compact wavefunction is key for the fast evaluation of observables on the ground state.

We presented a DMC extrapolation scheme that can be applied to great effect within the framework of the SHDMC iterative procedure. Our procedure to extrapolate DMC results to the infinite determinant limit achieves total energies in close agreement (1.3(7) mHa) with the CBS extrapolated selected CI reference value. This procedure might be useful to study with DMC complex systems beyond the reach of quantum chemistry approaches.

Finally, we have investigated the asymptotic compactness of the SHDMC wavefunction in comparison with selected CI wavefunction (cc-pVQZ orbital basis). We showed that the SHDMC procedure not only results in significantly more compact wavefunction in absolute terms, but that the exponential rate of convergence to the infinite determinant limit is increased, suggesting that larger systems may be approached with SHDMC before reaching the ultimate exponential-scaling wall faced by all known exact electronic structure methods.

The relatively minimal computational resources utilized demonstrate that for simple, but nonetheless interesting systems, SHDMC calculations can be performed in a resource constrained environment. Since vastly superior computational resources, such as the Frontier and Aurora exascale supercomputers, are accessible, the SHDMC method can readily be tested and validated in future applications to much larger and more challenging systems.

In order to apply SHDMC to more complex 2D materials and other strongly correlated materials in the future, alternative methods of constructing an initial multideterminant trial wavefunction are needed. Due to the steep scaling of computational cost with respect to system size displayed by existing methods such as selected CI, current work on developing methodology to efficiently find a high-quality active without depending on computationally costly quantum chemistry methods is in progress.

Because all FN-DMC calculations were performed in a single two atom unit cell at the $\Gamma$ point, it is important to acknowledge that finite size effects impact the results, especially because the long-range correlations contributing to the ground state energy may not be sufficiently accounted for^59^. While in the future SHDMC calculations could be performed for larger cells, a small cell allows us to estimate the impact of short range correlations. Future work will assess the accuracy of a supercell correction approach where SHDMC provides a short range energy shift on top of single determinant DMC performed in larger supercells. Additional future work will focus on generalizations of SHDMC to complex wavefunctions^60^ in solids to enable the twist averaging^61^ needed to reduce finite size error in applications to larger and more complex materials. The availability of advanced computational resources, combined with efficient methodology for constructing a reasonable multideterminant trial wavefunction and the use of twist averaging, will enable the application of SHDMC to 2D materials and other highly correlated solids.

## Supplementary Information

Supplementary Information.

## Data Availability

The data used to generate all of the figures and tables presented in this manuscript has been uploaded to the Constellation dataset repository and is available at https://doi.ccs.ornl.gov/dataset/8b36ddb5-3fbd-5985-a2e8-2c193ab48fff and are available from the corresponding author on reasonable request.
