# Supplementary material for: Multireference diffusion Monte Carlo reaches 2D materials
Source: Sci Rep. 2025 Sep 26;15:32984. doi: 10.1038/s41598-025-17564-3 (PMC12475236; doi:10.1038/s41598-025-17564-3)
Supplement: Supplementary file 1 — Supplementary Information. [file 41598_2025_17564_MOESM1_ESM.pdf]

Supplementary Information for "Multireference Diffusion Monte Carlo Reaches 2D Materials"

Nicole Spanedda,[†] Anouar Benali,[‡] Fernando A. Reboredo,[†] and Jaron T. Krogel[*,†]

†*Materials Science and Technology Division, Oak Ridge National Laboratory*

‡*Argonne Computational Science and Material Science Divisions, Argonne National Laboratory*

E-mail: spaneddans@ornl.gov,reboredofa@ornl.gov,krogeljt@ornl.gov

In Table S1 we compare the total energies obtained from SHDMC with T-moves and with the locality approximation (SHDMC-LA-QZ) with sCI variational energies (E_{var}) and rPT2 corrected sCI energies (E+rPT2). All of the SHDMC results were obtained using the cc-pVQZ atomic basis set, while sCI results were obtained for a sequence of correlation consistent atomic basis sets.

In Figure S1, we display the results of a time-step extrapolation performed on the converged SHDMC:Locality Approximation and SHDMC:T-moves wavefunctions, obtained in the 20,000 determinant active space (see section 4.3 in main paper). In Figure S1 the DMC energies in the limit of timestep $= 0\text{Ha}^{-1}$ for the extrapolations labeled SHDMC-QZ-TM and SHDMC-QZ-LA were found to be $-11.19446 \pm 5.7776 \times 10^-5$ and $-11.19481 \pm 6.0630 \times 10^-5$, respectively.

In Figure S2 a plot of energy vs. the square root of cumulative computational cost (random initial trial wavefunction) reveals that two different rates of convergence occur, during the SHDMC iterative procedure, when starting from the random initial trial wavefunction.

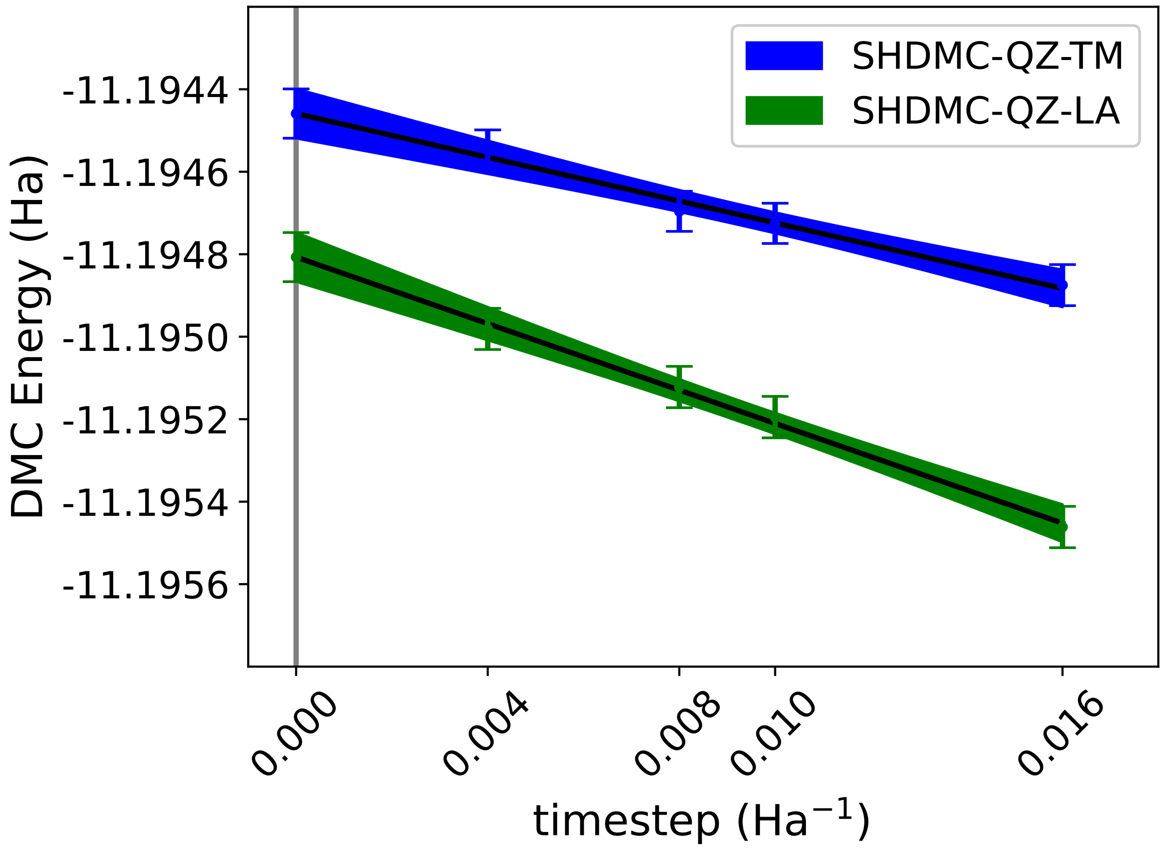

Figure S1. Timestep extrapolation with the converged SHDMC wavefunction obtained in the 20,000 determinant basis. Extrapolations based on this single wavefunction are shown both for DMC with T-moves (SHDMC-QZ-TM, blue) and the locality approximation (SHDMC-QZ-LA, green). The error bars indicate the error in the Monte Carlo estimate of the the energy (error in the DMC energy). Using the calculated errorbars shown in the figure, we resampled the dataset (5,000 samples) and performed total least squares linear fits for each sample. The mean of each fit appears as solid lines on the plot, while the standard error of each fit is shown in either blue SHDMC-QZ-TM, blue) or green (SHDMC-QZ-LA, green).

Table S1. Raw data: DMC and CIPSI results

Method	Total Energy (Ha)	Error (Ha)	No. Dets
SHDMC:Locality Approx.	-11.19481	$\pm$ 1.8 $\times 10^{-5}$	8994
SHDMC:T-Moves	-11.19446	$\pm$ 2.8 $\times$ 10^{-5}	8994
sCI Basis	E+rPT2 (Ha)	E_{var} (Ha)	No. Dets
cc-pVDZ	-11.082223	-11.082180	1.7×10^6
cc-pVTZ	-11.165237	-11.165105	13.9×10^6
cc-pVQZ	-11.188444	-11.188163	25.0×10^6
cc-pV5Z	-11.194059	-11.193464	14.6×10^6
cc-pV6Z	-11.196475	-11.194964	3.5×10^6

Comparison between total energies (in units of Hartrees), obtained from SHDMC with T-moves (SHDMC-TM-QZ) and with the locality approximation (SHDMC-LA-QZ), sCI variational energies (E_{var}) and rPT2 corrected energies (E+rPT2), obtained using atomic basis sets provided in the column labeled sCI basis. The SHDMC-TM-QZ and SHDMC-LA-QZ total energies are the average local energies (with statistical re-blocking) obtained in the limit of timestep = 0 Ha^{-1}, using the converged SHDMC wavefunction as the trial wavefunction. The errors in the SHDMC-TM-QZ and SHDMC-LA-QZ total energies are the statistical errors in the estimate of the average local energy (DMC total energy). The number of determinants, with a nonzero weight, used to construct each multideterminant wavefunction is provided in the column labeled No. Dets. Both the SHDMC-TM-QZ and SHDMC-LA-QZ wavefunctions were obtained using a 20,000 determinant active space.

During the initial iterations of SHDMC a faster rate of convergence is observed, followed by a slower rate of convergence during the later iterations of the SHDMC process. The faster rate of convergence during the initial SHDMC iterations, observed in Figure S2, is consistent with the conclusions drawn in Ref. [1] and by examining the curve labeled SHDMC:Random in Figure 1d in the main text.

When starting from the truncated CI initial trial wavefunction, the rate of convergence of the total energy with respect to the square root of the cumulative computational cost, of the iterative SHDMC procedure, is best represented by two different regimes (Figure S3). In Figure S3, the initially large error bars and fluctuations in the total energy, during the first 10 iterations of the SHDMC procedure, is generally expected when starting from a high-quality wavefunction. Due to the small number of configurations sampled at the beginning of the SHDMC procedure, fluctuations in the wavefunction are mostly driven by statistical error. In contrast to what is observed when starting from a poor-quality initial trial wavefunction,

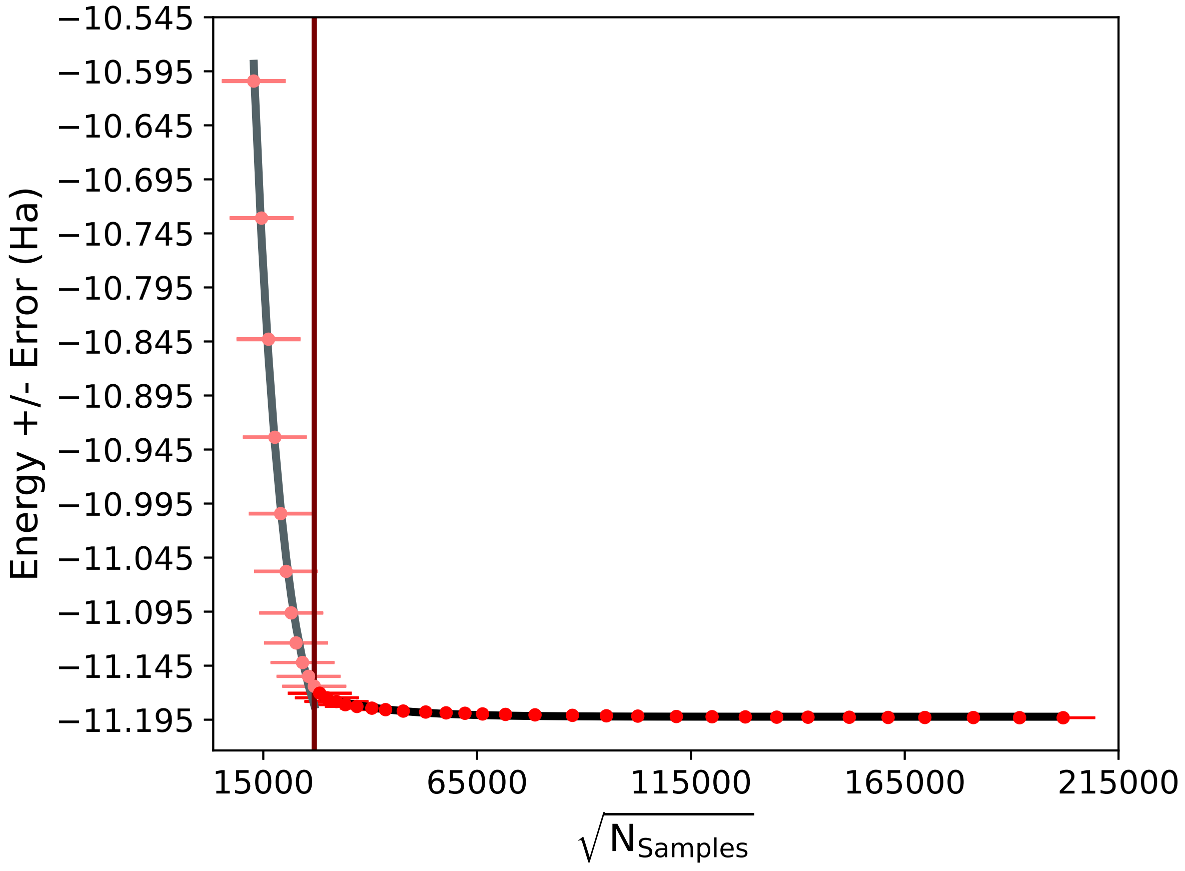

Figure S2. Rate of energy convergence: Random initial trial wavefunction. Plot of energy vs. square root of cumulative computational cost reveals two distinct rates of convergence. The rate of convergence observed during the initial iterations of SHDMC (left of vertical line) is accurately modeled by the decaying exponential $y = 4.501e^{(-1.486\times10^{-4})x} - 11.264$ with a variance weighted coefficient of determination of $R^2 = 0.994$. The rate of convergence observed during the later iterations of SHDMC (right of vertical line) is accurately modeled by the decaying exponential $y = 0.148e^{(-7.213\times10^{-5})x} - 11.192$ with a variance weighted coefficient of determination of $R^2 = 0.978$. The error bars indicate the error in the Monte Carlo estimate of the the energy (error in the DMC energy)

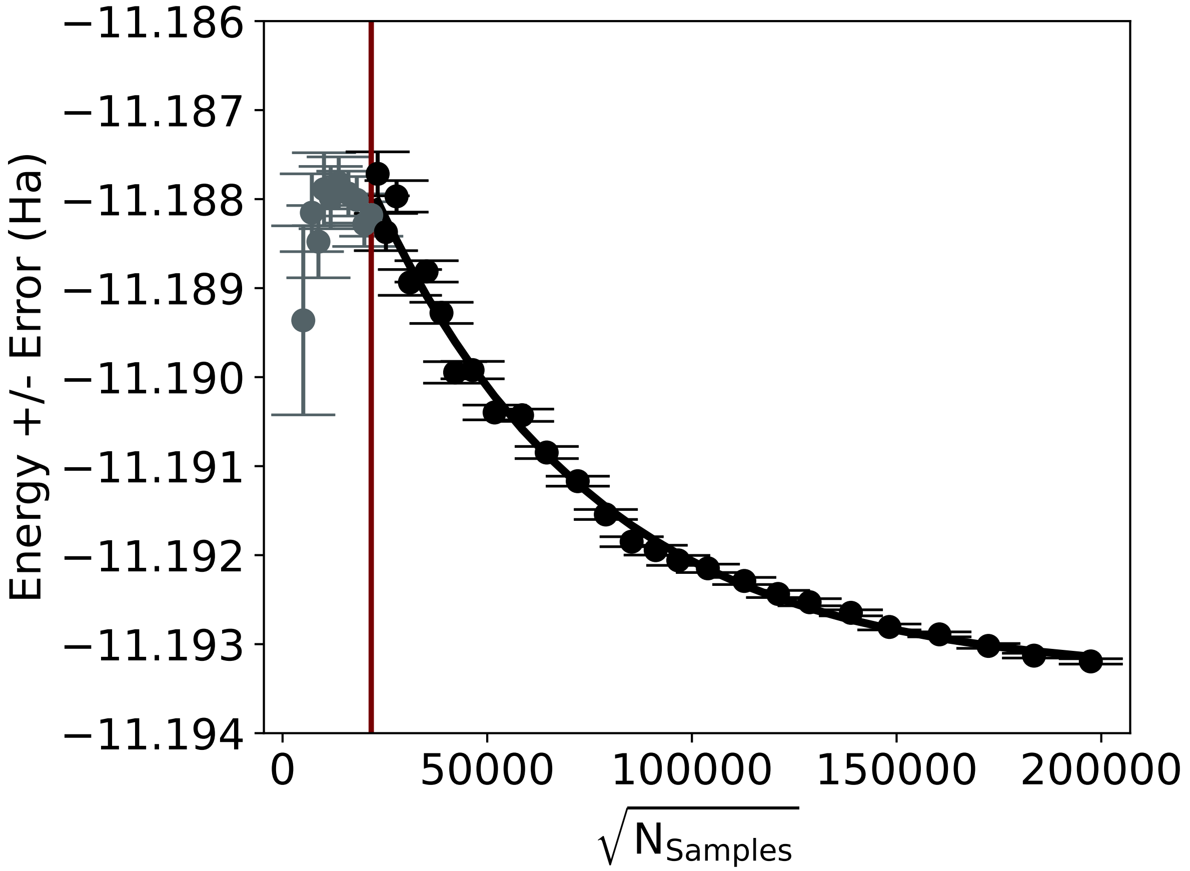

Figure S3. Rate of energy convergence: Truncated sCI initial trial wavefunction. Plot of Energy vs. square root of cumulative computational cost indicates two different convergence regimes. The results obtained from the first ten iterations of SHDMC (left of vertical line) are dominated by noise and the rate of convergence does not seem to be well represented by a meaningful or distinct functional form. The rate of convergence observed for the remaining iterations (right of vertical line) is accurately modeled by the decaying exponential $y = 8.401 \times 10^{-3} e^{(-1.976 \times 10^{-5})x} - 11.193$ with a variance weighted coefficient of determination of $R^2 = 0.984$. The error bars indicate the error in the Monte Carlo estimate of the the energy (error in the DMC energy)

this lesser sampling effort causes an initial decrease in the quality of the wavefunction. By examining Figure S3 it can be observed that as the sampling effort increases the quality of the wavefunction surpasses that of the initial truncated CI trial wavefunction.

By comparing Figure S2 and Figure S3 it can be observed that although a higher cumulative computational cost is required to achieve convergence when starting from the random initial trial wavefunction, the rate of convergence is faster when starting from a poor-quality initial trial wavefunction compared to the rate of convergence achieved when starting from a high-quality intial trial wavefunction. The noise driven fluctuations in the wavefunction that occur during the initial iterations can actually contribute to improving the quality of the wavefunction, when starting from a poor-quality initial trial wavefunction.

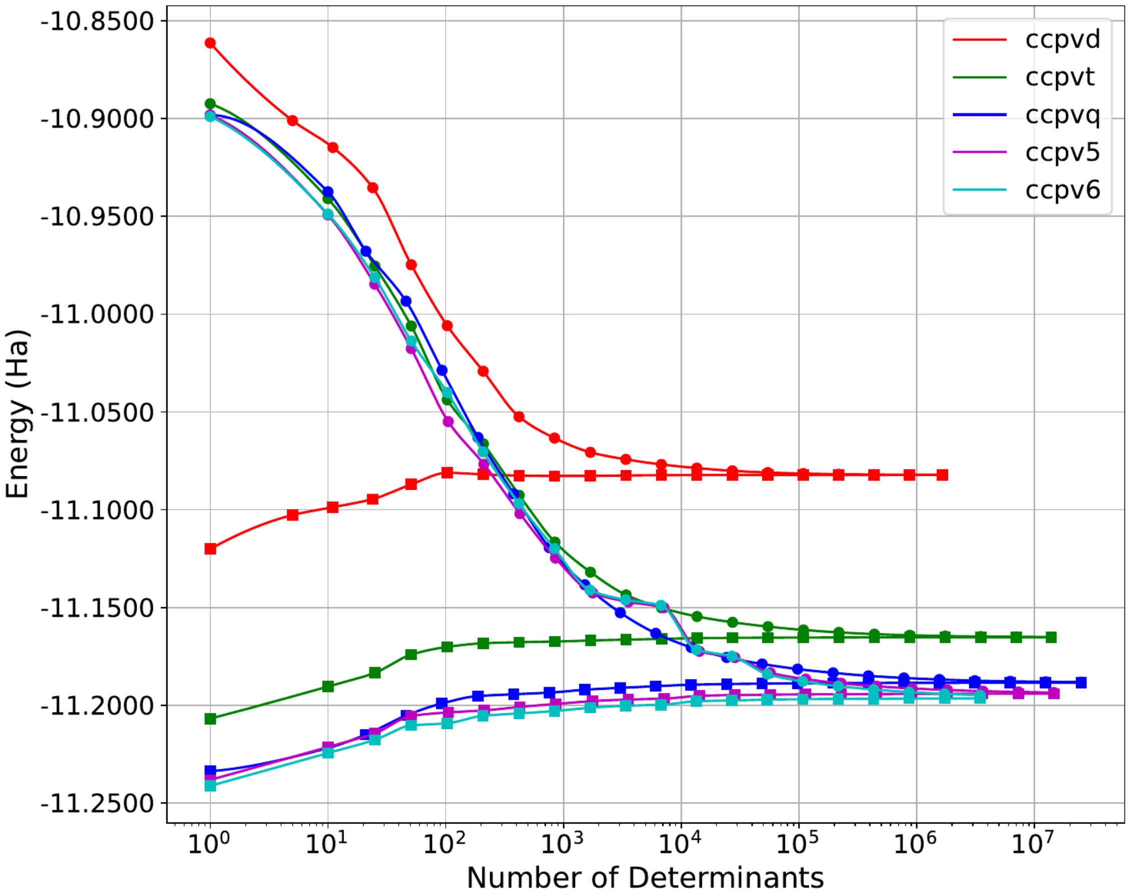

Figure S4. Convergence of selected CI energies for graphene. This figure demonstrates the convergence of selected CI energies for graphene, using cc-pVDZ to cc-pV6Z atomic basis sets, as additional determinants are added iteratively. Variational total energies (in units of Hartrees) are shown with solid circles while rPT2 corrected energies appear as solid squares. The lines serve as guides to the eye.

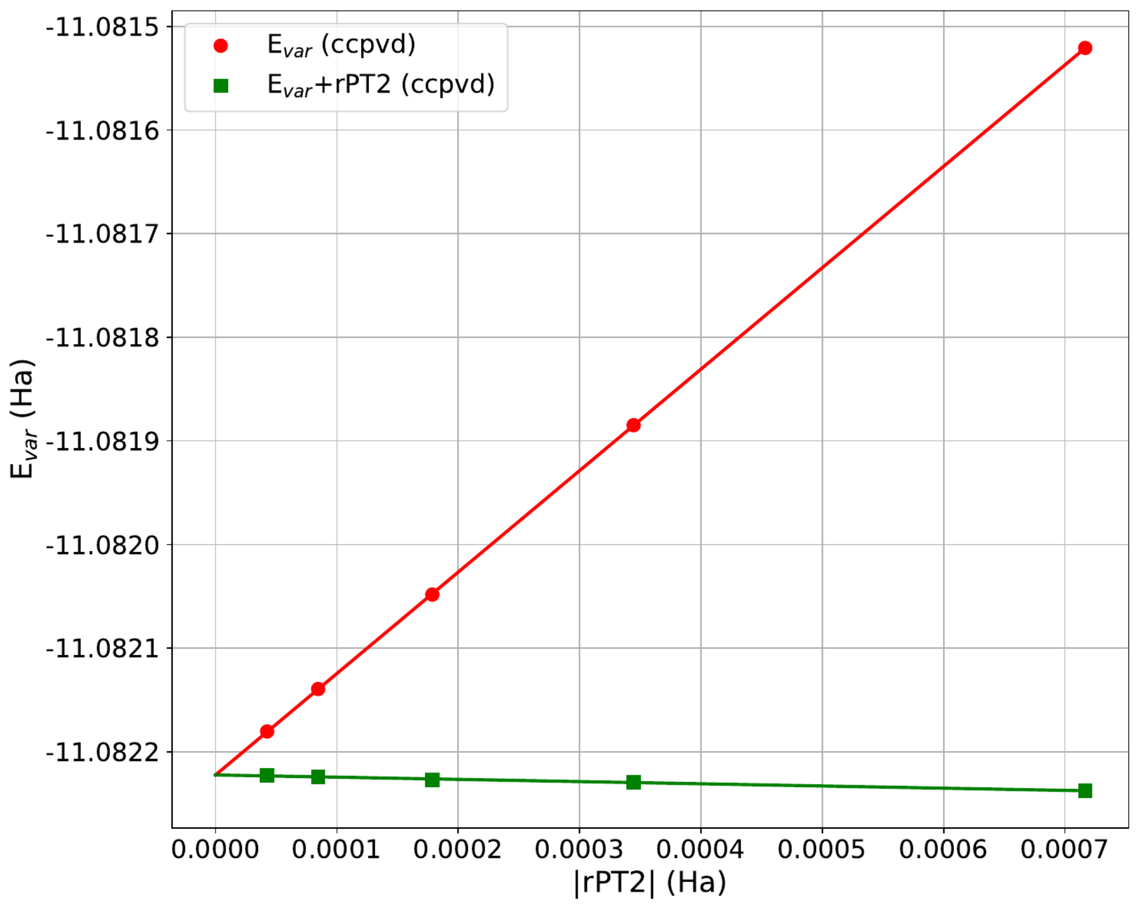

Figure S5. Extrapolation of selected CI cc-pVDZ energies to the infinite determinant limit. The variational energies (E_{var}) are shown as solid circles and the rPT2 corrected energies ($E_{var} + rPT2$) in solid squares, and are plotted versus the magnitude of the rPT2 correction ($|rPT2|$). The solid lines are linear fits to the data, with the estimate of the energy at the infinite determinant limit appearing at the intersection point of the two lines, where the rPT2 correction to the energy goes to zero.

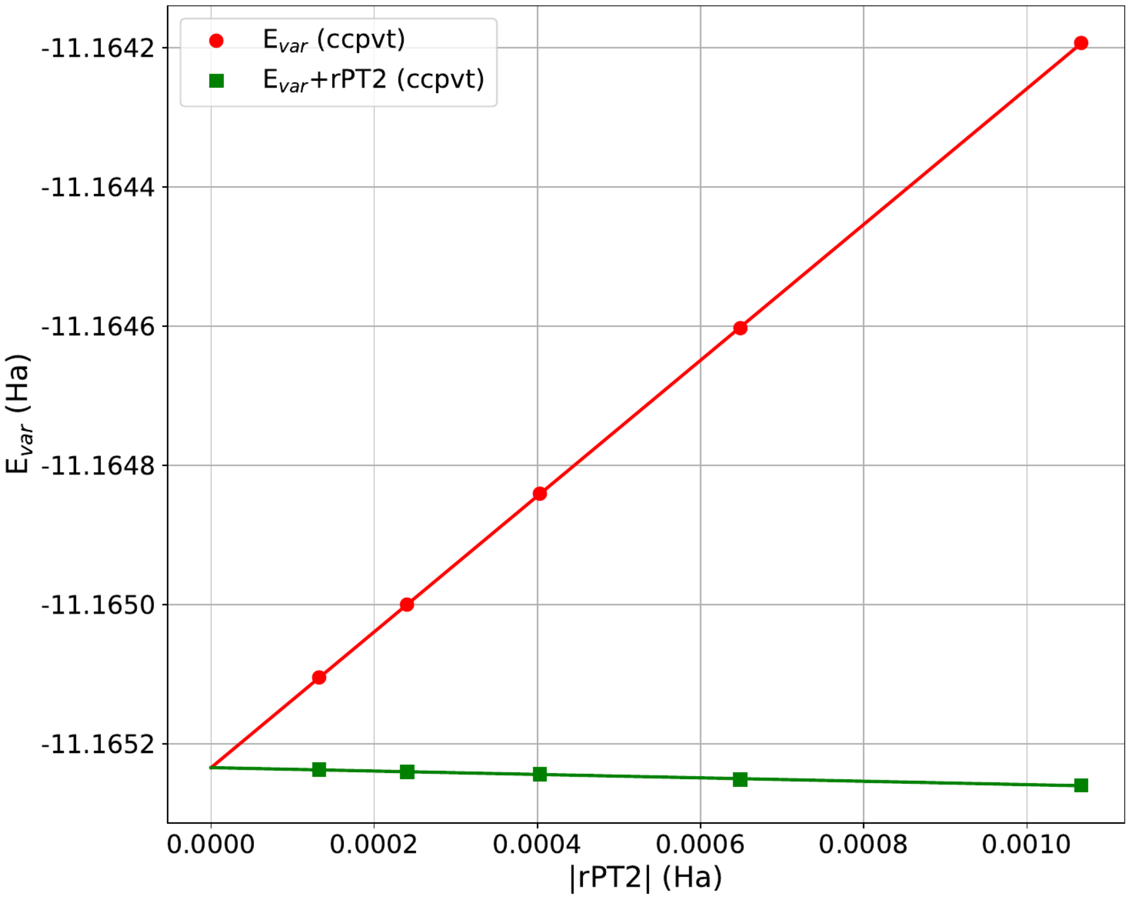

Figure S6. Extrapolation of selected CI cc-pVTZ energies to the infinite determinant limit. Variational energies (E_{var}) and rPT2 corrected variational energies ($E_{var} + rPT2$), which are represented by are represented by solid circles and solid squares, respectively, are plotted versus the magnitude of the rPT2 correction ($|rPT2|$). The solid lines are linear fits to the data, with the estimate of the energy at infinite determinant limit appearing at the intersection point of the two lines, where the rPT2 correction to the energy goes to zero.

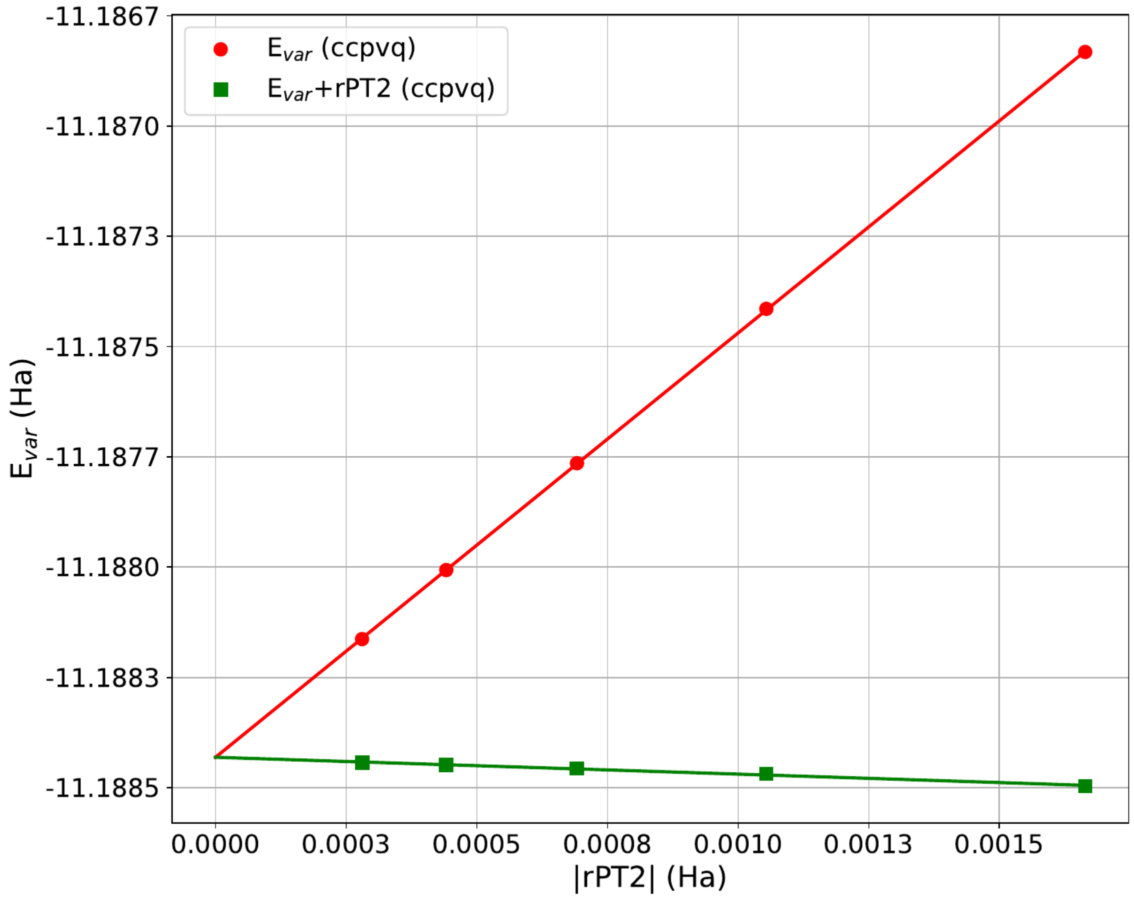

Figure S7. Extrapolation of selected CI cc-pVQZ energies to the infinite determinant limit. The variational energies (E_{var}) are shown as solid circles and rPT2 corrected variational energies ($E_{var} + rPT2$) in solid squares and are plotted versus the magnitude of the rPT2 correction ($|rPT2|$). The solid lines are linear fits to the data, with the estimate of the energy at the infinite determinant limit appearing at the intersection point of the two lines, where the rPT2 correction to the energy goes to zero.

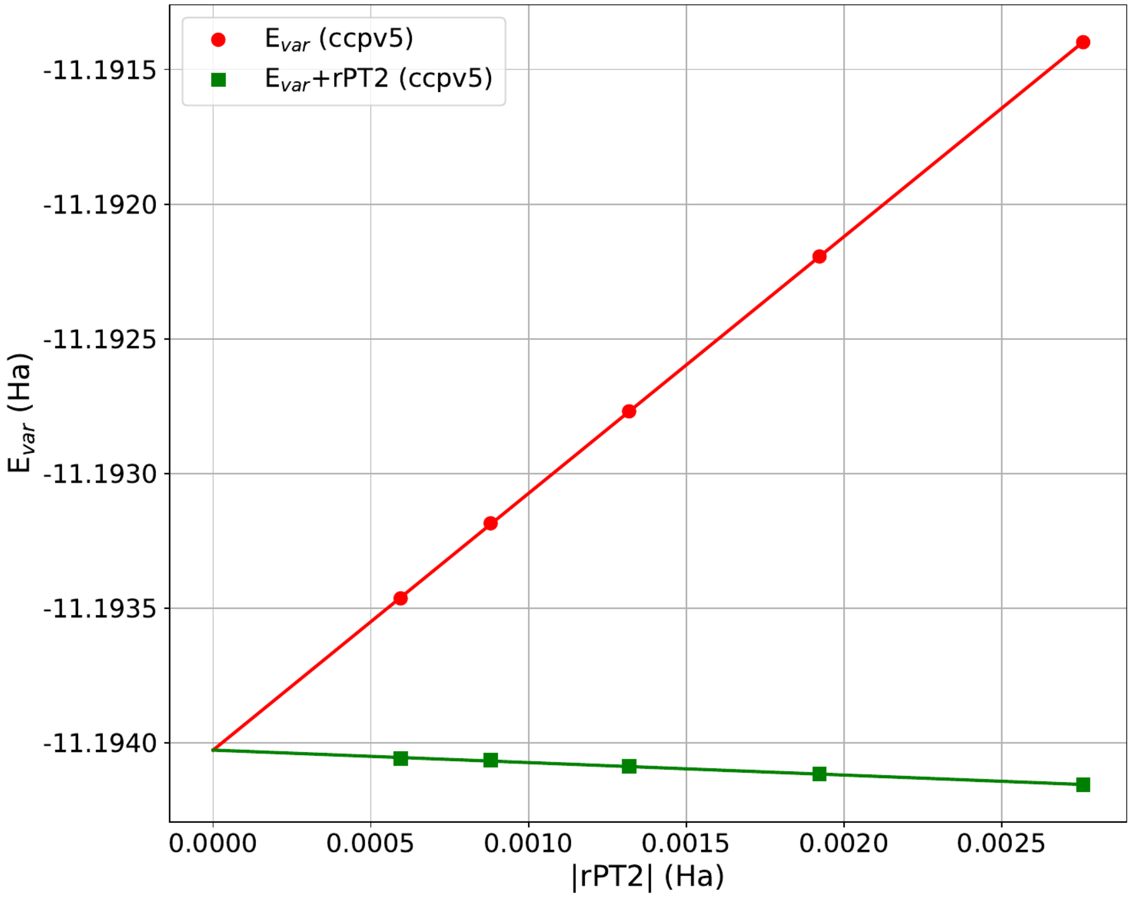

Figure S8. Extrapolation of selected CI cc-pV5Z energies to the infinite determinant limit. Variational energies (E_{var}) are shown as solid circles and rPT2 corrected variational energies ($E_{var} + rPT2$) in solid squares and are plotted versus the magnitude of the rPT2 correction. The solid lines are linear fits to the data, with the estimate of the energy at the infinite determinant limit appearing at the intersection point of the two lines, where the rPT2 correction to the energy goes to zero.

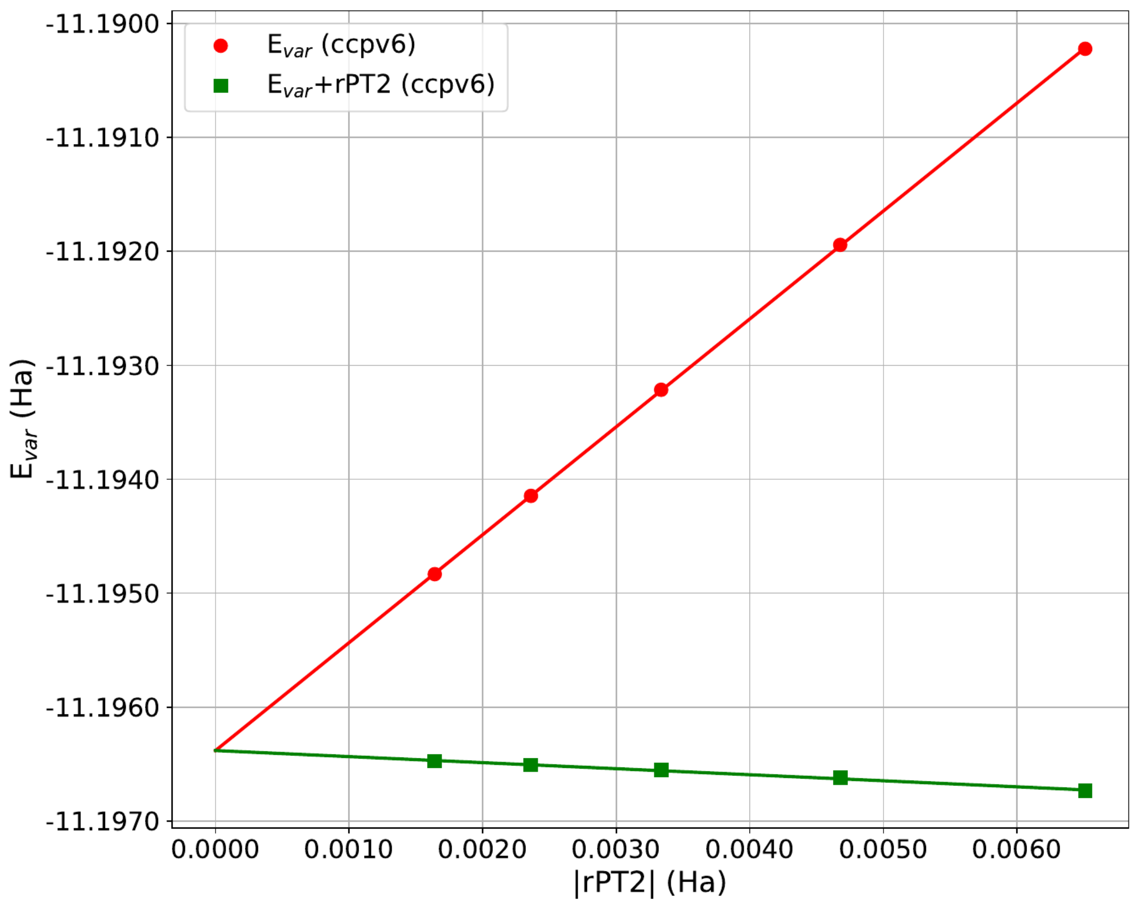

Figure S9. Extrapolation of selected CI cc-pV6Z energies to the infinite determinant limit. Variational energies (E_{var}) are shown as solid circles and rPT2 corrected energies ($E_{var} + rPT2$) in solid squares and are plotted versus the magnitude of the rPT2 correction ($|rPT2|$). The solid lines are linear fits to the data, with the estimate of the infinite determinant limit appearing at the intersection point of the two lines, where the rPT2 correction to the energy goes to zero.

Table S2. Lattice vectors for graphene primitive unit cell

a	b	c
4.032461	0.000000	0.000000
-2.328143	4.656285	0.000000
0.0000000	0.000000	37.794522

Lattice vectors (bohr) for graphene primitive unit cell provided to QMCPACK input file.

Table S3. Atomic positions (2-carbon atom unit cell)

Element	x	y	z
C	0.000000	0.000000	18.897261
C	1.344154	2.328143	18.897261

Atomic positions provided to QMCPACK input file in Cartesian coordinates.

References

[1] F. A. Reboredo, R. Q. Hood, and P. R. C. Kent. "Self-healing diffusion quantum Monte Carlo algorithms: Direct reduction of the fermion sign error in electronic structure calculations". In: Phys. Rev. B 79 (19 May 2009), p. 195117. DOI: 10.1103/PhysRevB.79.195117. URL: https://link.aps.org/doi/10.1103/PhysRevB.79.195117.
